# Notch activation is required for downregulation of HoxA3-dependent endothelial cell phenotype during blood formation

**DOI:** 10.1371/journal.pone.0186818

**Published:** 2017-10-26

**Authors:** Valentina Sanghez, Anna Luzzi, Don Clarke, Dustin Kee, Steven Beuder, Danielle Rux, Mitsujiro Osawa, Joaquín Madrenas, Tsui-Fen Chou, Michael Kyba, Michelina Iacovino

**Affiliations:** 1 Division of Medical Genetics, Department of Pediatrics, Harbor-UCLA Medical Center, Torrance, CA, United States of America; 2 Los Angeles Biomedical Research Institute, Torrance, CA, United States of America; 3 Lillehei Heart Institute, University of Minnesota, Minneapolis, MN, United States of America; 4 CiRA | Center for iPS Cell Research and Application, Kyoto University, Kyoto, Japan; 5 Department of Microbiology and Immunology, McGill University, Montréal, QC, Canada; Feinberg Cardiovascular Research Institute, Northwestern University, UNITED STATES

## Abstract

Hemogenic endothelium (HE) undergoes endothelial-to-hematopoietic transition (EHT) to generate blood, a process that requires progressive down-regulation of endothelial genes and induction of hematopoietic ones. Previously, we have shown that the transcription factor HoxA3 prevents blood formation by inhibiting Runx1 expression, maintaining endothelial gene expression and thus blocking EHT. In the present study, we show that HoxA3 also prevents blood formation by inhibiting Notch pathway. HoxA3 induced upregulation of Jag1 ligand in endothelial cells, which led to *cis*-inhibition of the Notch pathway, rendering the HE nonresponsive to Notch signals. While Notch activation alone was insufficient to promote blood formation in the presence of HoxA3, activation of Notch or downregulation of Jag1 resulted in a loss of the endothelial phenotype which is a prerequisite for EHT. Taken together, these results demonstrate that Notch pathway activation is necessary to downregulate endothelial markers during EHT.

## Introduction

Hematopoietic stem cells (HSC) originate in the Aorta-Gonad-Mesonephros (AGM) region of the embryo [1–4]. A subset of endothelial cells lining the aorta undergoes changes to generate the hematopoietic clusters that line the ventral portion of the aorta [5, 6]. This process is known as endothelial-to-hematopoietic transition (EHT). EHT requires coordinated downregulation of endothelial lineage genes and upregulation of hematopoietic lineage genes, and involves interaction between endothelial cells and stroma cells surrounding the aorta [7, 8].

We have shown that HoxA3, a transcription factor of the homeodomain family [9, 10], blocks EHT by directly inhibiting Runx1 expression [11]. Such a blockade correlates with maintenance of endothelial cell phenotype and no appearance of hematopoietic cell phenotype. Thus, EHT likely involves a signaling pathway that downregulates endothelial cell gene expression and orchestrates the acquisition of hematopoietic cell phenotype [11]. During the search of candidate signaling pathways that regulate EHT, we noted that Jag1 was significantly upregulated in HE. The Notch pathway is activated when a Notch ligand such as Dll1, Dll3, Dll4, Jag1, or Jag2 interacts in *trans* with Notch receptor 1 to 4 on an adjacent cell. Upon this interaction, the intracellular domain of the receptor is cleaved and translocated to the nucleus where it activates the CSL complex, resulting in the induction of Notch target genes [12]. Activation of the Notch pathway becomes more complex when ligands and receptors are both expressed on the same cell because, under those conditions, ligands can inhibit the receptors through a phenomenon called *cis*-inhibition [13–16].

Activation of the Notch pathway is necessary for proper vasculogenesis and angiogenesis *in vivo* [17–19] and for blood formation [20–23]. In addition, the requirement of the Notch pathway for HSC formation varies depending on the stages of EHT. The pathway is absolutely required at the beginning of EHT and it becomes dispensable when HSC are fully formed [24]. This is corroborated by the observation that mice lacking Jag1 do not develop blood [22]. It has been recently proposed that Jag1 functions to counteract Dll4 activity during blood development [25]. While the studies described above address the necessity of the pathway for blood formation, it is unclear how regulation of Notch receptors and ligands affect pathway activation or repression and ultimately EHT.

Here, we identify a new mechanism by which HoxA3 tightly controls blood formation by inhibiting the Notch pathway in HE. We show that HoxA3 induced up-regulation of Jag1 in HE that has not yet started blood commitment. This upregulation caused Notch ligand *cis*-inhibition and blocked the initiation of blood commitment. Consistent with our model, forced activation of Notch pathway, either in a ligand-independent fashion or by down-regulation of Jag1, triggered blood commitment by down-regulating the endothelial cell phenotype, a step required for the initiation of EHT. These findings suggest that HoxA3 prevents blood commitment through Notch *cis*-inhibition and identify the Notch pathway as a potential therapeutic target for blood development *in vitro*.

## Materials and methods

### Cell culture

HoxA3-inducible murine ES cells were previously generated, by cassette exchange recombination in doxycycline-inducible locus [11]. ES cells were co-cultured with MEFs in knockout DMEM supplemented with 15% FBS, 0.1 mM nonessential amino acids, 2 mM glutamax, penicillin/streptomycin, 0.1 mM β-mercaptoethanol, and 1000 U/mL LIF, at 37°C in 5% CO_2_. Embryoid Bodies (EBs) were differentiated by hanging-drops suspension culture in EB differentiating media (IMDM supplemented with 15% FBS, 200 μg/mL iron-saturated transferrin, 4.5 mM monothiolglycerol, 50 μg/mL ascorbic acid, penicillin/streptomycin, and 2 mM glutamax at 37°C in 5% CO_2_, 5% O_2_). At day 4 of differentiation, EBs were treated with doxycycline (1μM) to induce HoxA3 overexpression. At day 6, EBs were collected, dissociated by using trypsin at 37°C for 2–3 min following mechanical dissociation, and immunostained with Flk1-PE and Ve-cadherin-APC antibodies (S1 Table). Two hundred thousand double-positive cells were sorted by flow cytometry using FACs Aria III and plated on OP9 stromal cell monolayers (50,000 OP9 cells per well pre-plated in 6-well dishes one day prior) in IMDM supplemented with 10% FBS, 5 ng/mL VEGF, 40 ng/mL TPO, 40 ng/mL Flt-3 ligand, penicillin/streptomycin, 2 mM glutamax, with or without doxycycline at 37°C in 5% CO_2_. Notch inhibition was performed with DAPT treatment (20 and 60 μM). For experiments involving Notch ligand overexpression, OP9-Dll1 [26] and OP9 control cells were growth-arrested with Mytomicyn-c treatment (7.5 μg/ml) for 4 hours. OP9 growth arrested cells were plated a day before co-culture in fresh OP9 media. After co-culture in OP9, cells were harvested by trypsinization for 1 minute at 37°C followed by antibody staining.

### Plasmids

The pMSCV-hNICD-Ires-GFP was constructed by subcloning a blunted Not1/Ecor1 hNICD (from NOTCH1 receptor) fragment [27] into pMSCV-ires-GFP. For knock-down experiments, pLKO-Jag1 KD (clone TRCN0000028869, Open Biosystem) was used. Puromycin resistance was replaced with eGFP using blunt-end cloning.

### Retroviral/Lentiviral transduction

Retroviral transduction of pMSCV-ires-GFP, pMSCV-hNICD-ires-GFP and pLKO-Jag1 KD were performed as reported [11]. Retrovirus and lentivirus were generated by transient transduction of 293T with BioT reagent (Bioland). Supernatant was collected at 24, 48 and 72h after transfection and virus concentrated with a Sorvall RC6+ (rotor F21S-8X50y) by ultracentrifugation for 3 hours at 47.000g. Retroviral and Lentiviral transduction were performed on day 6 FV sorted progenitors and BEND3 cells.

### Quantitative real time RT-PCR (qRT-PCR)

Total RNA was extracted with Trizol LS (Invitrogen) and cDNA generated with iScript reagent (BioRad) according to protocol. PCR was performed using TaqMan mix (Life Technologies) on a 7600 RT-PCR System from Applied Biosystems. Probes purchased from Applied Biosystems are listed in S1 Table.

### Antibody staining

Flow cytometry was performed by staining with specific anti-mouse antibodies. Analysis was performed using FACS ARIA III and data analyzed using FlowJo software. For Immunofluorescence staining, cells were fixed in 4% PFA for 4 min following by antigen retrieval in Tris-EDTA buffer pH 9.0 and permeabilized as reported in 0.2% triton for 15 min [11]. Cells were incubated overnight with primary antibodies Jag1, VE-Cadherin ad activated-NICD. Pictures were acquired using 20X magnitude lens on Leica TCS SP Upright Confocal Microscope and 40X lens on Nikon Eclipse Ci fluorescent microscope. All antibodies used are listed in S1 Table.

### Western blot

293T cells were transfected with pMSCV-hNICD-iresGFP or pMSCV-iresGFP, proteins were extracted with RIPA Buffer (Millipore # 20–188). After blotting membranes were stained with Ponceau S and probed with anti-Myc-tag for NICD detection. Cytoplasmic proteins from Bend3 cells transfected with pLKO Jag1-KD or pGIPZ-GFP and Day 6 FV progenitors were extracted using NE-PER extraction kit (Thermo-Scientific #78833). After protein separation and blotting (Millipore #456–1096 and # 170–4158) membranes were probed using Jag1 specific antibody followed by specific secondary antibody. Membranes were imaged using BioRad Chemi Doc Imaging system. All antibodies used are listed in S1 Table.

### Statistical analysis

Data were analyzed using GraphPad Prism software (La Jolla California, USA). Data obtained from 6 hours, 5 days of HoxA3 up-regulation experiments and DAPT treatment on the expression of Notch target genes in BEND3 cells were analyzed with unpaired, two-tailed t-test. Independent data sets were analyzed by two-way ANOVA using HoxA3 regulation (Con vs HoxA3) and treatment (DMSO/DAPT, Con/NICD transduction, OP9 Con/ OP9 Dll1) as between-subject followed by Fisher’s LSD post hoc tests. One-way ANOVA was performed to analyze the effect of Jag KD on Con and HoxA3 upregulation. Significance was set at p<0.05.

## Results

### HoxA3 upregulates Jag1 but does not activate the Notch pathway

**Fig 1:**
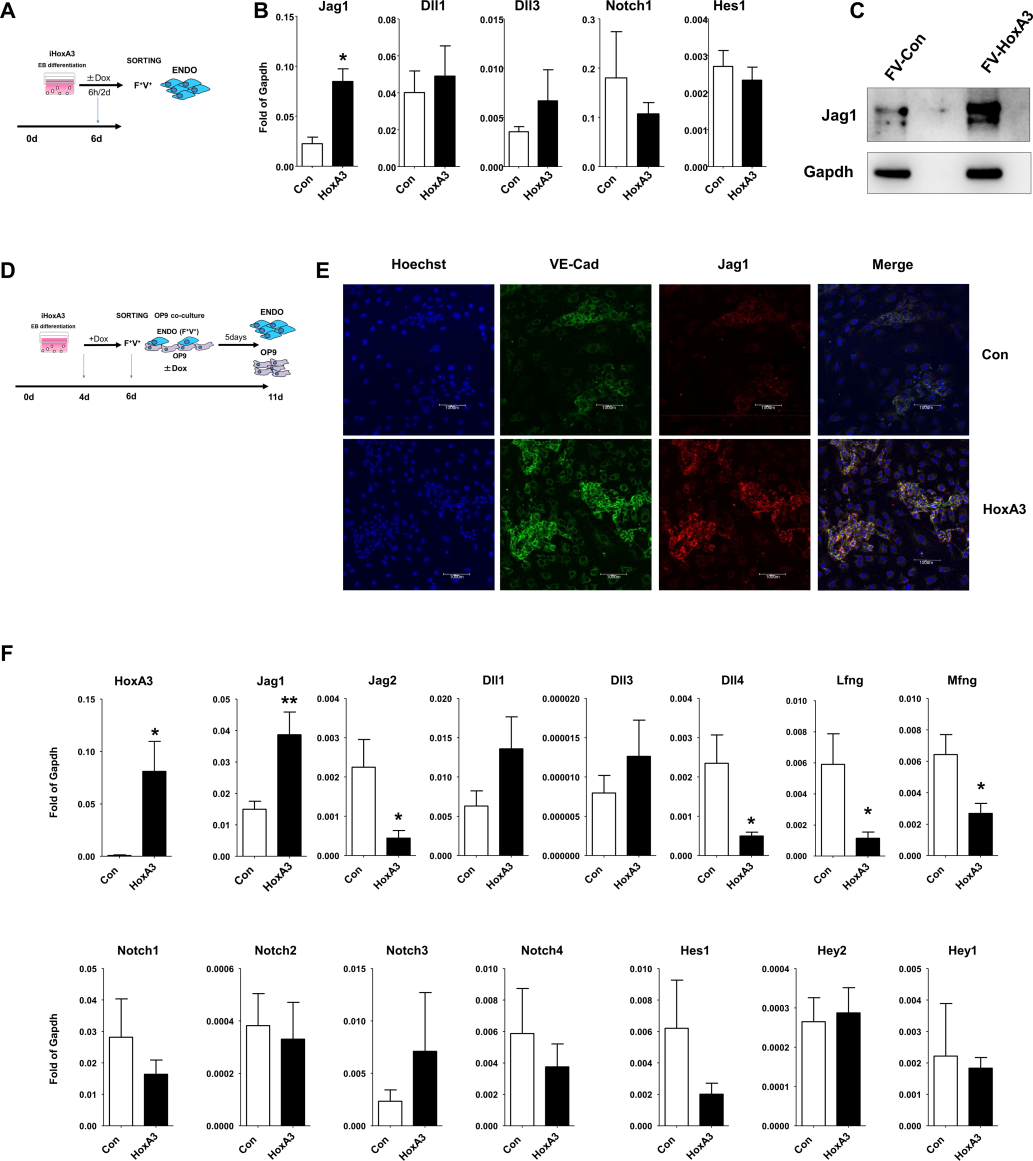
HoxA3 up-regulates the Notch ligand Jag1 but does not activate the Notch pathway. **A)** Experimental procedure (see methods for details). **B)** Gene expression levels of the Notch pathway components in purified endothelial cells (Flk1^+^/VE-Cadherin^+^) derived from day 6 control EBs (white bars) or upon 6 hours of HoxA3 up-regulation (black bars). **C)** Western blot analysis using Jag1 specific antibody in purified endothelial cells (Flk1^+^/VE-Cadherin^+^) derived from day 6 control EBs (white bars) or upon 2 days of HoxA3 up-regulation (black bars) **D)** Experimental procedure (see methods for details). **E)** Immunofluorescence staining for Jag1 (red), VE-Cadherin (green) and Hoechst (blue) showing adherent endothelial clusters growing in Control (Con) or HoxA3 overexpression (HoxA3), derived from endothelial cells (Flk1^+^/VE-cadherin^+^) and co-cultured for 5 days on OP9 cells. Bar: 100 μM. **F)** Gene expression levels of Notch ligands Jag1/Jag2/Dll1/Dll3/Dll4, Notch receptors Notch1 to Notch4, cofactors Lfng (lunatic fringe) Mfng (manic fringe) and target genes Hes1/Hey2/Hey1 on purified control cells (white bars) or cells overexpressing HoxA3 (black bars) derived from endothelial cells (Flk1^+^/VE-cadherin^+^) co-cultured for 5 days on OP9 cells. *: p<0.05. Detailed statistical analysis is reported in **S2 Table**.

We have previously reported an experimental model that recapitulates blood formation from EHT using embryonic stem cell differentiation. This model takes advantage of the ability of HoxA3 to block blood formation from HE, providing a synchronized and homogeneous population of HE [11] (S1A Fig). Under these conditions, we performed transcriptional analysis in HE upon brief HoxA3 up-regulation [11] (Fig 1A). In this analysis, we found that the Notch ligand Jag1 was upregulated 2.5 fold after a 6 hours induction of HoxA3, suggesting that HoxA3 controls Jag1 expression. Other Notch ligands such as Dll1 and Dll3, the Notch1 receptor, and the Notch target gene Hes1 were not upregulated (Fig 1B).Two days induction of HoxA3 resulted in strong upregulation of Jag1 at protein levels (Fig 1C). This result was unexpected since the effect of these molecules on blood formation is opposite: HoxA3 blocks blood formation [11] whereas Jag1 promotes blood formation [22]. This paradox was not due to a selective effect of HoxA3 on vein commitment nor its action on arterial commitment as there were no changes in the expression of the arterial markers CXCR4, CD44 and Efnb2, or in the expression of venous markers Ephb4 in the endothelial compartment (S1B and S1C Fig).

Next, we determined if the up-regulation of Jag1 expression by HoxA3 correlated with activation of the Notch pathway. This was done by culturing the HE on OP9 stroma cells for a longer period of time (Fig 1D). Taking advantage of the property of HoxA3 to repress blood formation during EB differentiation, we enriched HE in the endothelial compartment and modeled EHT *in vitro* using the OP9 co-culture. The starting population of endothelial cells expressed CD31 and Tie2 (not shown) as well as Flk1 and Ve-cadherin (S1A Fig). When the HE cells were grown on OP9, and upon downregulation of HoxA3, EHT occurred and blood was formed. However, if HoxA3 overexpression was maintained, then the HE cells remained in their endothelial state (S1D Fig) [11].

Under these conditions, we were able to evaluate the function of the Notch pathway during EHT. In the following experiments, we co-cultured HoxA3 induced cells during EB differentiation on OP9 for 5 days with or without HoxA3 up-regulation, then separated the EB-derived cells from the OP9 and performed qPCR analysis. Upon HoxA3 induction, both Jag1 mRNA levels and protein were significantly up-regulated in endothelial cells (Fig 1E and 1F). As shown by immunofluorescence (Fig 1E), Jag1 expression was present in every endothelial cell when HoxA3 was upregulated. Jag2 and Dll4 were significantly down-regulated whereas Dll1 and Dll3 were not. While there were no significant changes in the Notch receptors, the glycosyltransferase enzymes manic (Mfng) and lunatic (Lfng) fringe were downregulated (Fig 1F). These enzymes increase the affinity of Notch receptors to Dll1 ligand in *trans* [28] and thus, their downregulation is associated with lower ability of the endothelial cells to receive Notch signals. Under the same conditions, the Notch target genes Hey2, Hey1 and Hes1 showed no apparent activation in endothelial cells (Fig 1F). Such lack of activation was not due to the inability of HE cells to respond to Notch signals [24] but rather to the inability of OP9 to efficiently promote Notch activation [23, 29].

To test whether the increased expression of Jag1 in endothelial cells had a biological effect in *trans*, we evaluated the expression of Notch target genes in the OP9 stromal cells. We found that there was induction of the Notch pathway as measured by Hey2 upregulation only when OP9 cells were co-cultured with endothelial cells overexpressing HoxA3, (S1E Fig). Together, these data suggested that HoxA3 induces Jag1 up-regulation in endothelial cells which in turn induced Notch activation in *trans* (i.e., on OP9 cells) but not in *cis* (i.e., on endothelial cells).

### Repression of EHT by HoxA3 is not affected by inhibition of the Notch pathway

**Fig 2:**
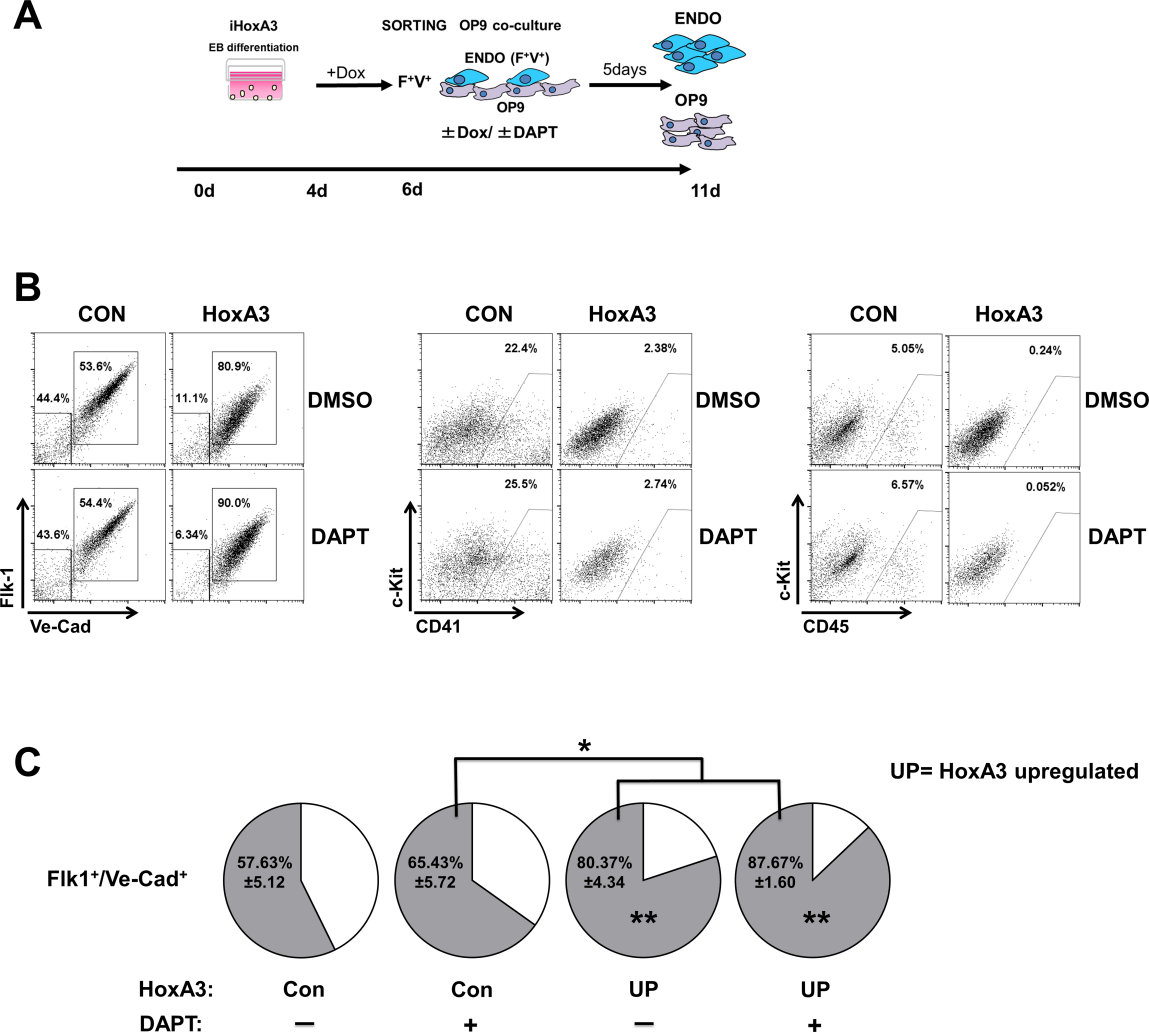
Repression of EHT by HoxA3 is not affected by inhibition Notch pathway. **A)** Experimental procedure, Endothelial Derived Cells (ENDO). **B)** Representative flow cytometric profile of endothelial surface markers Flk-1/Ve-Cadherin and hematopoietic surface markers c-Kit/CD41, and c-Kit/CD45 on 200,000 EB-derived Flk1^+^/VE-cadherin^+^ cells without or with HoxA3 overexpression and co-cultured on OP9 for 5 days in the presence or absence of the Notch inhibitor DAPT (20 μM). **C)** Frequency of endothelial surface markers Flk-1^+^/Ve-Cadherin^+^ in EB-derived cells. *: p<0.05; **: p<0.01. Two way ANOVA analyses of Flk-1^+^/Ve-Cadherin^+^, CD41^+^ and CD45^+^ frequencies are reported on **S3 Table**.

Next, we tested whether the blockade of blood formation was secondary to HoxA3-dependent Notch activation. To test this possibility, we differentiated EBs and plated them on OP9 cells with or without HoxA3 up-regulation and in the presence or absence of the Notch pathway inhibitor DAPT (Fig 2A). As expected up-regulation of HoxA3 resulted in repression of blood formation as measured by the lack of CD41 and CD45-expressing cells (Fig 2B, S2 Fig, gating strategies). Notch pathway inhibition was not able to restore blood formation in cells overexpressing HoxA3 nor did result in any changes in control cells (Fig 2B, S3A Fig) while still repressing efficiently Notch target genes (S3B Fig). A higher DAPT concentration had the same effect (data not shown). In addition, Notch inhibition did not affect the differentiation into myeloid cells (i.e., cells expressing CD45/Gr1) nor the arterial commitment of the cells (i. e., Ve-Cadherin^+^/Cxcr4^+^ and Ve-Cadherin^+^/CD44^+^) (S3C Fig). More importantly, DAPT treatment significantly increased the frequency of Flk1^+^ Ve-cadherin^+^ endothelial cells (Fig 2C). These data showed that Notch inhibition by itself cannot promote blood formation, ruling out the possibility that the blockade of blood formation is due to HoxA3-dependent Notch activation.

### Notch activation down-regulates the endothelial phenotype of HE but does not promote blood formation

**Fig 3:**
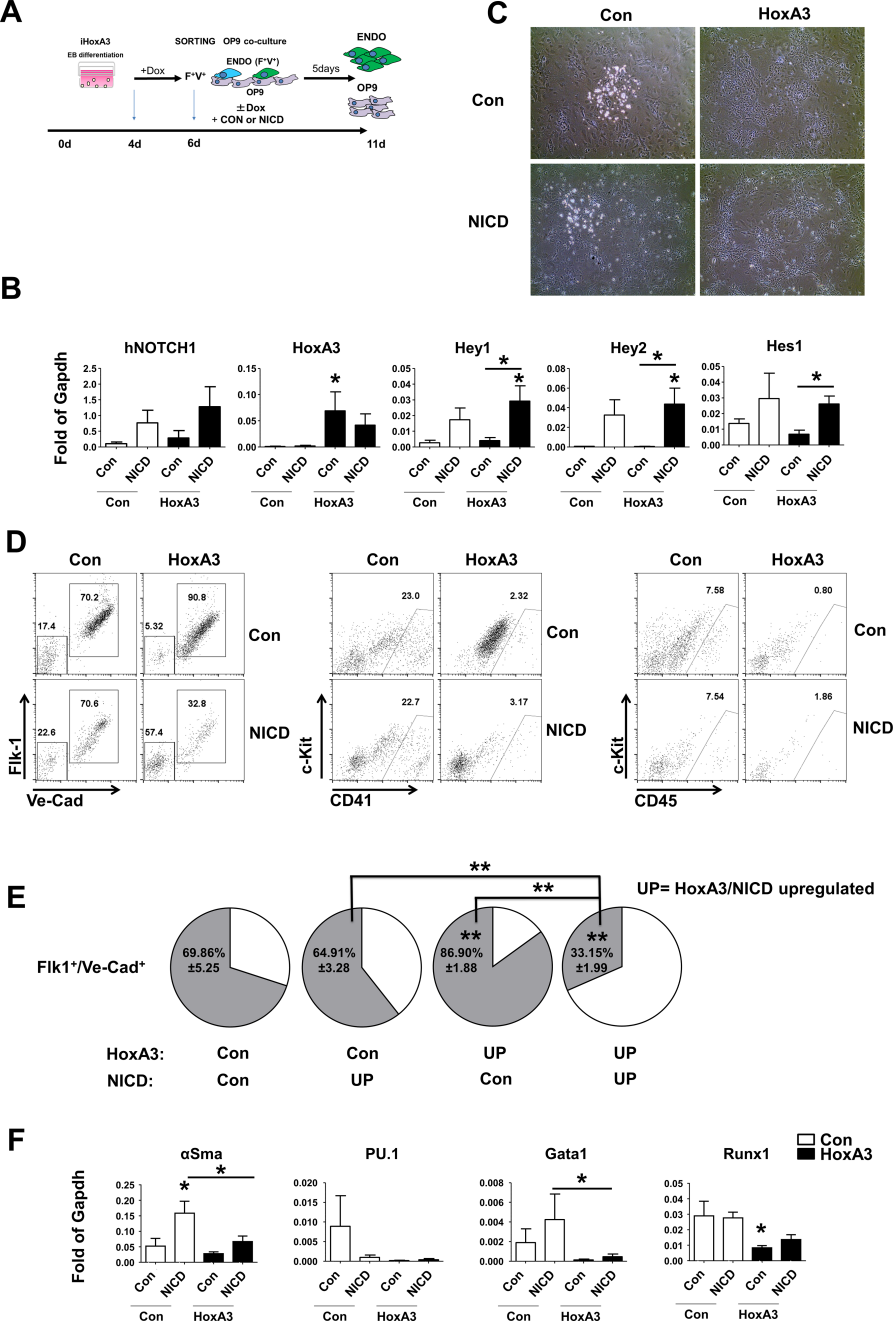
Notch activation down-regulates the endothelial phenotype of the hemogenic endothelium but does not promote blood formation. **A)** Experimental procedure, Endothelial Derived Cells (ENDO). **B)** Gene expression levels in purified NICD-transduced, GFP-positive cells co-cultured with OP9 for 5 day without or with doxycycline-induced HoxA3 overexpression. HoxA3, Notch pathway components hNotch1, Hes1, Hey1, Hey2 are shown. **C)** Representative images of 200,000 Flk1^+^/VE-cadherin^+^ cells obtained from day 6 EBs, transduced with pMSCV-iresGFP (CON) or with pMSCV-hNICD1-IresGFP and co-cultured on OP9 for 5 days in absence (CON) or presence (HoxA3) of HoxA3 overexpression. **D**) Representative flow cytometric profile of endothelial surface markers Flk-1/Ve-Cadherin and hematopoietic surface markers c-Kit/CD41, and c-Kit/CD45 of GFP+ gated cells from the same culture as in **B, C**. **E)** Frequency of endothelial surface markers Flk-1^+^/Ve-Cadherin^+^. **F)** Gene expression levels as in **B** of hematopoietic gene markers, Pu.1, Runx1, Gata1, and smooth muscle actin on GFP+ control (white bars) or HoxA3-induced (black bars) GFP+ cells are plotted. *: p<0.05. Statistical analysis is reported on **S4 Table**.

Next, we tested whether the blockade of blood formation was due to HoxA3-dependent repression of Notch activation. To do this, we forced activation of Notch in a ligand-independent fashion using NICD transduction (Fig 3A). NICD up-regulation was assessed by immunoblotting (S4A Fig) and by the expression of Notch target genes Hey1, Hey2 and Hes1 (Fig 3B). Hematopoietic clusters were visible in control and NICD-transduced group but could not be detected in NICD-transduced group when HoxA3 was overexpressed (Fig 3C). Thus, the blockade of blood formation itself was not due to HoxA3-dependent inhibition of Notch.

Blood formation involves not only induction of hematopoietic gene expression but also down-regulation of endothelial gene expression. Therefore, we next examined the effect of Notch activation on the endothelial phenotype. Induction of the Notch pathway in cells overexpressing HoxA3 resulted in a significant decrease in the frequency of Fkl1^+^/Ve-Cadherin^+^ endothelial cells (Fig 3D and 3E). Analysis of the frequency of PECAM expression, an additional endothelial marker, showed the same decrease (S4B Fig). Surprisingly, the decrease in frequency of endothelial cells did not correspond with an increase of hematopoietic cells as shown by CD41, CD45 (Fig 3D, S4C Fig), Ter119 and Gr1 expressing cells (S4D Fig), which would be expected if EHT had occurred. Therefore, the decrease in endothelial cell phenotype was not due to blood formation. The lack of blood formation in presence of NICD was not a consequence to increased apoptosis of precursor cells (S4E Fig) nor was due to differentiation of HE into smooth muscle cells. In fact, while up-regulation of Notch pathway resulted in differentiation into vascular smooth muscle cells in control cells [30] (measured as αSMA Fig 3F), this differentiation was inhibited in HoxA3 overexpressing cells. The lack of blood cells was rather due to inability of Notch signaling to overwrite HoxA3 function and induce expression of key hematopoietic transcription factors. In fact, when HoxA3 was up-regulated, hematopoietic markers Runx1, Pu.1 and Gata1 were still significantly down-regulated and the activation of the Notch pathway did not induce their expression (Fig 3F). Thus, we concluded that HoxA3 inhibits Notch pathway activation in HE and this maintains the endothelial cell phenotype.

### Notch signaling in trans does not rescue HoxA3-mediated inhibition of Notch

**Fig 4:**
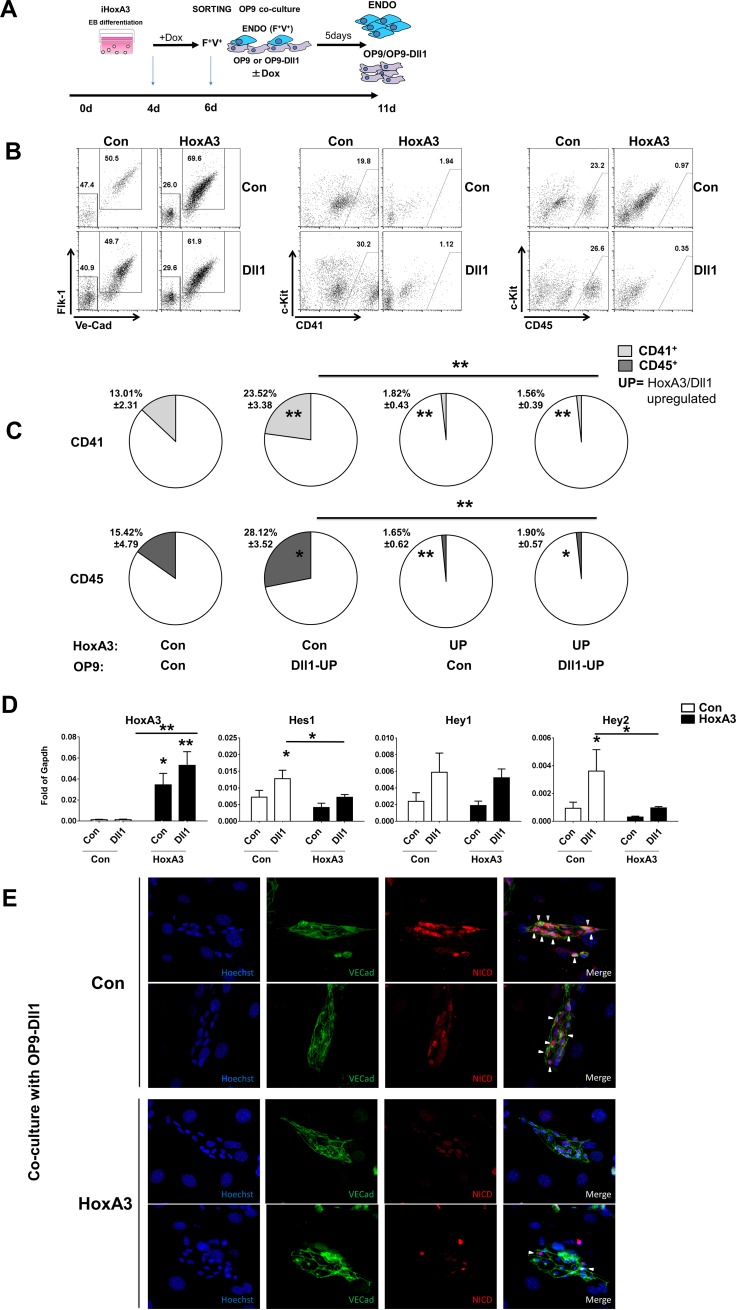
Notch signaling in *trans* does not rescue HoxA3 mediated inhibition of Notch. **A)** Experimental procedure **B)** Representative flow cytometric profile of endothelial surface markers Flk-1/Ve-Cadherin and hematopoietic surface markers c-Kit/CD41, and c-Kit/CD45 obtained from 200,000 EB-derived Flk1^+^/VE-cadherin^+^ cells and co-cultured on OP9 control (CON) or OP9 overexpressing Dll1 (OP9-Dll1) for 5 days in Control or HoxA3-overexpressing HE cells. **C)** Quantification of frequencies of hematopoietic surface markers (CD41, CD45) of the same cell as in **B**. **D)** Gene expression levels of the Notch pathway target genes (Hes1, Hey1, Hey2) and hematopoietic gene markers (PU.1, Runx1, Gata1) on control (white bar) or HoxA3-overexpressing (black bars) HE cells co-cultured on OP9 controls (CON) or OP9-DLL1 for 5 days (Flk1^+^/VE-cadherin^+^ and CD41^+^/c-Kit^+^ cells were pulled together). **E)** Iimmunofluorescence staining for activated Notch1 (NICD-red), VE-Cadherin (VECad-green) and Hoechst (blue) showing adherent endothelial clusters growing in Control (Con) or HoxA3 overexpression (HoxA3), derived from endothelial cells (Flk1^+^/VE-cadherin^+^) co-cultured on OP9-DLL1 cells *: p<0.05. Statistical analysis is reported on **S5 Table**.

Next, we examined whether Notch ligands in *trans* could activate Notch signaling in HoxA3-overexpressing cells. To test this hypothesis, we exposed HE to OP9 overexpressing the Notch ligand Dll1. After 5 days of co-culture, HE-derived cells were analyzed for blood formation and for Notch pathway activation (Fig 4A). As shown in Fig 4B and 4C, strong Notch signal in *trans* could not promote blood formation in the presence of HoxA3 overexpression as measured by the frequency of CD41^+^ and CD45^+^ cells, in contrast to control cells in which strong Notch signal could increase the frequency of hematopoietic cells (Fig 4C). As expected, no changes in the frequency of endothelial cell were observed (S5A Fig) suggesting that the Notch pathway could not be activated despite the availability of Notch ligands in *trans*. This possibility was corroborated by analysis of the expression of Notch target genes. Hes1 and Hey2 were induced by OP9-Dll1 in control cells and this induction was greatly diminished by HoxA3 overexpression (Fig 4D). Finally, we observed a decrease in the number of cells expressing nuclear NICD when HoxA3 was upregulated compared to control cells in the co-culture system using OP9-Dll1 (Fig 4E).

### Downregulation of Jag1 in HE prevents HoxA3-dependent Notch pathway cis inhibition

**Fig 5:**
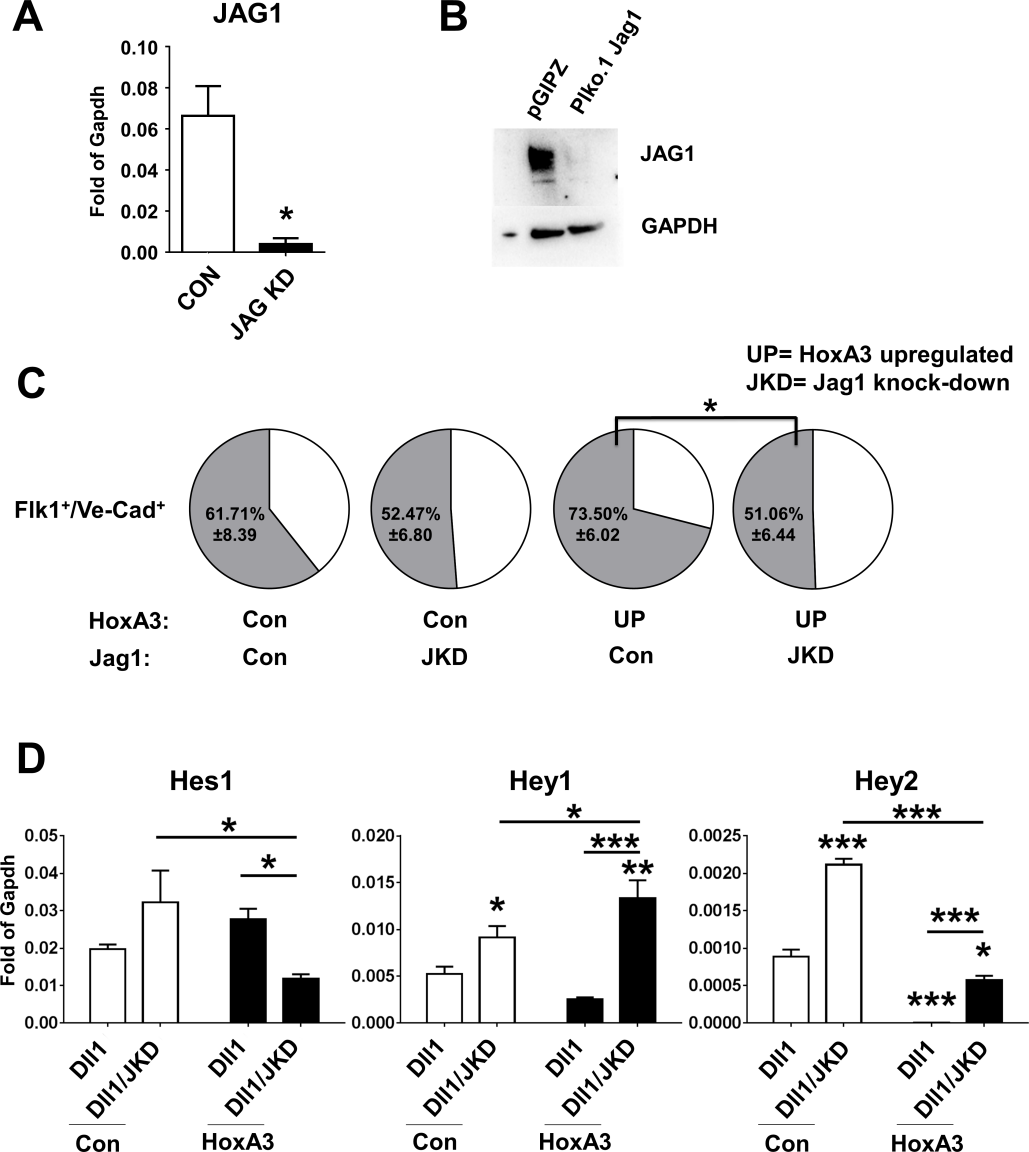
Forced down-regulation of Jag1 in endothelial cells removes Notch pathway cis inhibition by HoxA3 and initiates EHT. **A)** Jag1 RNA expression level in Bend3 cells infected with empty vector (Con) or Jag1 shRNA (Jag1 KD). **B)** Western blot using specific antibody against Jag1 and GAPDH in Bend3 cells infected with empty vector (pGIPZ) or Jag1 shRNA (Plko.1 Jag1). **C)** Frequency of Flk1+/VE-cadherin+ cells obtained from day 6 EBs, transduced with empty vector (CON) or with shRNA-Jag1-GFP (JKD) and co-cultured on OP9 for 5 days in Control (Con) or HoxA3 overexpression. **D)** Gene expression levels of Notch pathway components Hes1, Hey1, Hey2 from purified control (white bar) or HoxA3-overexpressing cells (black bars), transduced with empty vector (CON) or with shRNA-Jag1-GFP (JKD) and co-cultured on OP9-DLL1 for 5 days. Graphs in panel D show one representative experiment with triplicate measurements. *: p<0.05; **: p<0.01; ***: p<0.001. Statistical analysis is reported on **S6 Table**.

Since addition of Notch ligands in *trans* did not overcome the inhibitory effect of HoxA3 on the Notch pathway, we postulated that such inhibition was secondary to the presence of high levels of Jag1 on the HE that would then inhibit Notch signaling in *cis*. To test this possibility, we down-regulated Jag1 expression upon transduction of the cells with shRNA specific for Jag1. This resulted in a significant decrease in Jag1 expression both at mRNA level and protein level (Fig 5A and 5B). When HE cells were co-cultured on OP9, there was a significant down-regulation of the endothelial markers Flk-1 and Ve-Cadherin in the presence of HoxA3 overexpression (Fig 5C). Importantly, Jag1 down-regulation was not sufficient to trigger blood formation in control cells nor in HoxA3 overexpressing cells consistent with the claim that Notch determines the endothelial phenotype but does not drive by itself blood formation (S5B Fig). In addition, strong Notch signals in *trans* (Op9-Dll1 co-culture), while unable to induce blood formation (not showed), induced an increase of Notch target genes even if HoxA3 was overexpressed confirming that Jag1 up-regulation in HE was cis-inhibiting the Notch pathway (Fig 5D).

## Discussion

Cell to cell communication between the endothelium and the surrounding stromal cells is crucial for the generation of HSCs [8, 31, 32]. The Notch pathway plays a central role in this communication. It has been proposed that the stroma sends Notch signals to induce Runx1 [8, 20], which in the dorsal aorta marks the initiation of blood formation [33, 34]. We have previously shown that HoxA3 in the endothelium represses Runx1 expression and times the initiation of blood commitment [11]. In the current paper, we show that activation of Notch causes down-regulation of endothelial genes expression that is needed for EHT. More importantly, our data demonstrate that HoxA3 expression inhibits Notch pathway to maintain endothelial gene expression in the HE, preventing EHT. Such inhibition of the Notch pathway that maintains the endothelial phenotype of HE, results from a *cis* mechanism, i.e., up-regulation of Jag1 in these cells induced by HoxA3.

Our findings imply that the role of Notch is linked to regulation of the endothelial phenotype. We found that HE expresses all the components for Notch signaling and therefore is susceptible to regulation by this pathway. When HoxA3 is up-regulated there is a concomitant up-regulation of Jag1. The presence of this ligand on the same cells that express Notch receptors leads to down-regulation of Notch signaling and this maintains endothelial protein expression. In contrast, when HoxA3 is down-regulated, Jag1 expression is decreased and this relieves Notch *cis* inhibition, allowing cells to receive Notch signals required to initiate EHT. Such a claim is consistent with the observation that blocking Notch activity did not lead to an increase in blood formation [35]. Our findings are also in line with a report showing that Notch activation is required to proceed with the stage of EHT [24], a stage characterized by decreased HoxA3 expression and increased Runx1 expression.

Our results reveal *cis*-inhibition of the Notch pathway by high expression of the Notch ligand Jag1 on the responding HE as an important regulatory mechanism of EHT. Notch *cis*-inhibition was initially reported in Drosophila [16]. Our findings are consistent with a previous report proposing an inhibitory function of Jag1 in EHT [25]. In our system, we show that when Jag1 is downregulated, endothelial markers are downregulated, consistent with initiation of EHT, but this is not sufficient for blood formation. This fine mechanism may be sensitive to the timing of Jag1 deletion. In our experiment endothelial cells were fully developed prior downregulation of Jag1, while *in vivo* experiments, Jag1 was deleted throughout development [25, 36].

Since activation of the Notch pathway by strong Dll1 signal in *trans* is capable of promoting EHT but activation of Notch alone is not, we concluded that EHT needs activation of several Notch receptors achieved through signals delivered by Dll1 in *trans*. In contrast, Notch 1 alone would promote down-regulation of endothelial markers. In our experiments, the Notch pathway by itself was insufficient to promote EHT because HoxA3-dependent Runx1 repression, is dominant [1, 11, 37]. In other words, activation of the Notch pathway while Runx1 is inhibited promoted the HE to an intermediate stage in which the cells had lost their endothelial characteristics but were not able to acquire the hematopoietic signature.

**Fig 6:**
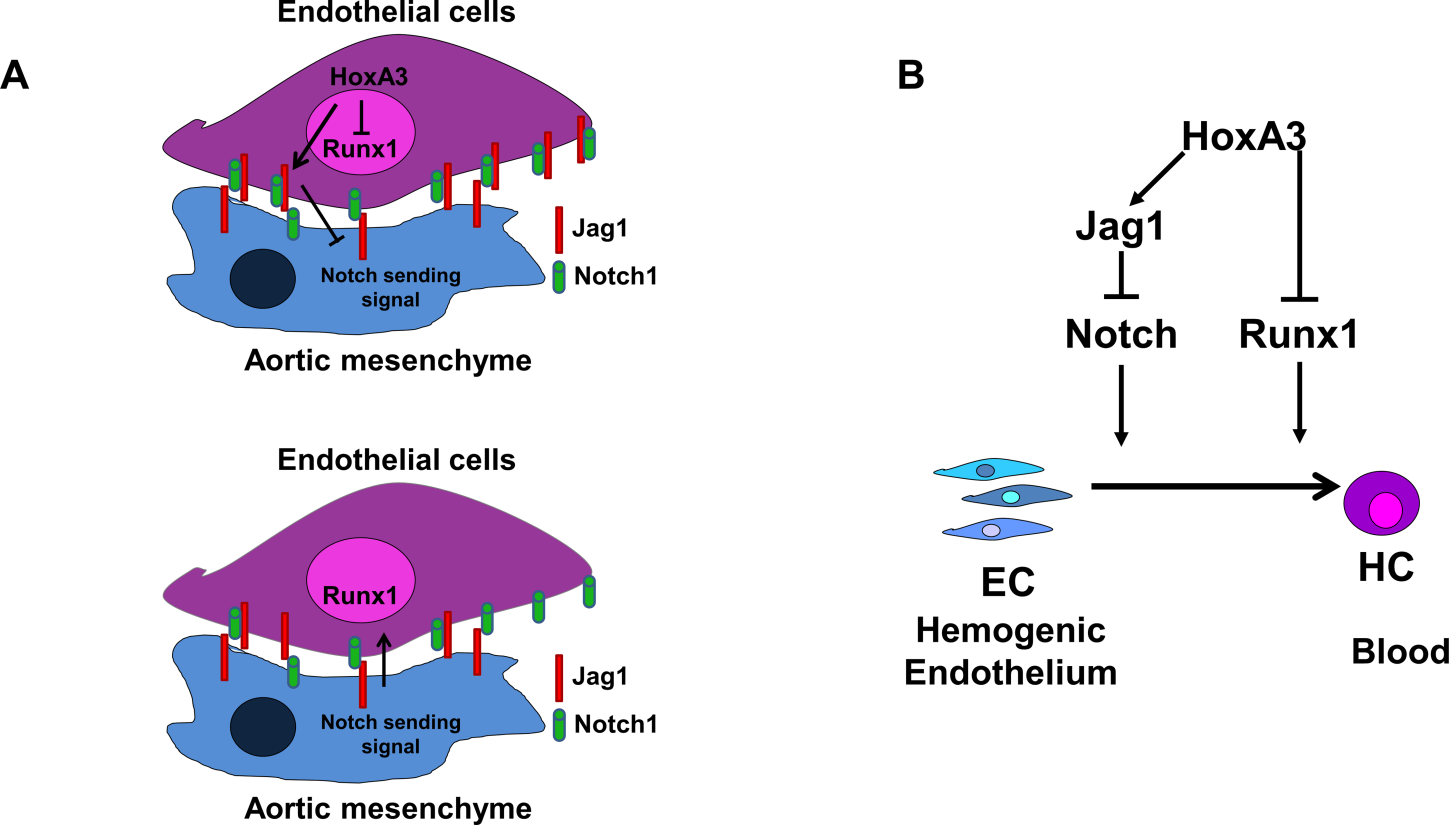
Proposed model. **A)** Illustration of ligand *cis* inhibition in the hemogenic endothelium. HoxA3 dependent Jag1 overexpression in *cis* interacts with Notch receptors, inhibiting Notch ligand in *trans* to interact with the receptor. When HoxA3 is withdrawal the pathway is activated in *trans* and Runx1 can be induced in the hemogenic endothelium. **B)** Effect of HoxA3 on Notch pathway. HoxA3 upregulates Jag1 to inhibit the Notch pathway. The pathway in turn will promote downregulation of endothelial specific transcripts, and initiate the EHT.

Based on our data, we propose a model to explain EHT as a regulated process triggered by proper communication between the HE and the stroma, and involving down-regulation of endothelial genes and up-regulation of the hematopoietic genes (Fig 6). In our model, when HoxA3 is up-regulated in the endothelium, it induces Jag1 expression on the same cells causing Notch ligand *cis* inhibition. This inhibition prevents cells from losing their endothelial phenotype. When HoxA3 is down-regulated, then Notch communication with the stroma can be properly established triggering down-regulation of endothelial markers and expression of hematopoietic markers, and this results into blood formation. Our model identifies HoxA3 as key regulator of Notch signaling and as a potential target for the modulation of blood formation.

## Supporting information

S1 Fig **A)** Representative flow cytometric profile of endothelial surface markers Flk-1/Ve-Cadherin and hematopoietic surface markers c-Kit/CD41, of 10000 cells obtained from dissociated day 6 EBs treated with or without Dox at day 4 of differentiation. **B)** Representative flow cytometric profile and frequency quantification of arterial surface markers Ve-Cadherin/CD44 and Ve-Cadherin/CXCR4 on 200,000 EB-derived Flk1+/VE-cadherin+ cells without or with HoxA3 overexpression and co-cultured on OP9 for 5 days. **C)** Gene expression levels in purified endothelial cells co-cultured with OP9 for 5 day without or with doxycycline-induced HoxA3 overexpression. Arterial, EfnB2 and vein markers EphB4 are plotted. **D)** Representative flow cytometric profile and frequency quantification of hematopoietic surface markers c-Kit/CD41, and c-Kit/CD45 on 200,000 EB-derived Flk1+/VE-cadherin+ cells without or with HoxA3 overexpression and co-cultured on OP9 for 5 days **E)** Assessment of Notch pathway activation on OP9 cells alone (left) or purified OP9 cells after co-culture with Flk1+/VE-cadherin+ without or with HoxA3 overexpression (right). Notch target genes Hes1 and Hey2 are plotted. Where present asterisks (*) identify significant paired two-tailed T test (* p<0.05). Statistical analysis is reported on **S2 Table**.
(PDF)

S2 Fig Representative flow-cytometric profile of PE and PECy7 isotype controls and CD41-PE and CD45- PECy7 markers of 200,000 cells Flk1+/VE-cadherin+ obtained from day 6 EBs and co-cultured on OP9 for 5 days in absence of HoxA3. (PDF)

S3 Fig **A)** Quantification of frequencies of hematopoietic surface markers (ckit-CD41, ckit-CD45) on 200,000 EB-derived Flk1+/VE-cadherin+ cells without or with HoxA3 overexpression and co-cultured on OP9 for 5 days in the presence or absence of the Notch inhibitor DAPT (20μM) **B)** Evaluation of Notch pathway inhibition (calculated as inhibition of Notch target genes Hes1, Hey1, Hey2, Hes6) on endothelial cells (BEND3) treated with 20μM of DAPT or DMSO (CON). **C)** Frequency quantification of 200,000 cells Flk1+/VE-cadherin+ obtained from day 6 EBs and co-cultured on OP9 for 5 days with or without HoxA3 overexpression and treated without (DMSO/CON) or with 20μM of DAPT. Hematopoietic surface markers Gr1-CD45 and arterial/vein Ve-Cadherin, CXCR4 and CD44 and are plotted. Statistical analysis is reported on **S3 Table**.
(PDF)

S4 Fig **A)** Western blot analysis and Ponceau S staining of the indicated proteins (cMyc-NICD and GAPDH) and total loading protein, respectively, in 293T cells transfected with pMSCV-hNICD-ires GFP plasmid (NICD-1/NICD-2), backbone vector pMSCV-ires GFP (CON) and non-viral infection (NVI). **B)** Frequency quantification of endothelial markers VeCadherin and Pecam (CD31), from gated GFP positive cells transduced with pMSCV-iresGFP (CON) or with pMSCV-hNICD1-IresGFP (NICD) and co-cultured on OP9 for 5 days in absence (CON) or presence (HoxA3) of HoxA3 overexpression. **C)** Quantification of frequencies of hematopoietic surface markers ckit, CD41, CD45, and **D)** representative flow cytometric profile of myeloid markers CD45, Gr1 and Ter119 on 200,000 cells Flk1+/VE-cadherin+ obtained from day 6 EBs, transduced with pMSCV-iresGFP (CON) or with pMSCV-hNICD1-IresGFP (NICD) and co-cultured on OP9 for 5 days in absence (CON) or presence (HoxA3) of HoxA3 overexpression. **E)** Frequency quantification and representative flow cytometric profile, of 200,000 cells Flk1+/VE-cadherin+ obtained from day 6 EBs, transduced with pMSCV-iresGFP (CON) or with pMSCV-hNICD1-IresGFP (NICD) and co-cultured on OP9 for 5 days in absence (CON) or presence (HoxA3) of HoxA3 overexpression. Viability markers PI and Annexin V are plotted. Post-hoc analysis are reported as asterisks (*) alone represents significant differences compared to CON/Dox-, * p<0.05, and bars represents significant differences (*) between indicated groups, p<0.05. Statistical analysis is reported on **S4 Table**.
(PDF)

S5 Fig **A)** Quantification of frequencies of endothelial surface markers Flk-1+/Ve-Cadherin+ obtained from 200,000 EB-derived Flk1+/VE-cadherin+ cells and co-cultured on OP9 control (CON) or OP9 overexpressing Dll1 (OP9-Dll1) for 5 days in Control or HoxA3-overexpressing HE cells. **B)** Quantification of frequencies of hematopoietic surface markers (cKit-CD41, cKit-CD45) on cells obtained from day 6 EBs, transduced with empty vector (CON) or with shRNA-Jag1-GFP (JKD) and co-cultured on OP9 for 5 days in Control (Con) or HoxA3 overexpression.
(PDF)

S1 Table Taqman probes, primary and secondary antibodies list. (PDF)

S2 Table Referred to Fig 1 and S1 Fig. **A)** Two tails T-test analysis of Notch components on control endothelial cells (CON) compare to endothelial cells derived from 6 hours upregulation of HoxA3 in D6 total EBs (HoxA3) **B)** Two tails T-test analysis of Notch components endothelial derived cells (EDC) co-cultured with OP9 for 5 days without (CON) or with HoxA3 overexpression.
(PDF)

S3 Table Referred to Fig 2 and S3 Fig. 2-way ANOVA analysis of endothelial derived cells co-cultured with OP9 for 5 days without (CON) or with HoxA3 overexpression and treated without (DMSO) or with (DAPT) Notch inhibitor.
(PDF)

S4 Table Referred to Fig 3 and S4 Fig. 2-way ANOVA analysis of endothelial derived cells transduced with pMSCV-NICD-ires GFP (NICD) or pMSCV-iresGFP (CON) and co-cultured with OP9 for 5 days without (CON) or with HoxA3 overexpression.
(PDF)

S5 Table Referred to Fig 4 and S5 Fig. 2-way ANOVA analysis on endothelial derived cells co-culture with OP9-CON vs OP9-Dll1 for 5 days without (CON) or with HoxA3 overexpression.
(PDF)

S6 Table Referred to Fig 5 and S5 Fig. 2-way ANOVA analysis of endothelial derived cells transduced with pGIPZ (CON) or plKO.1-Jag1 KD-ires GFP (JAG KD) and co-cultured with OP9 for 5 days without (CON) or with HoxA3 overexpression.
(PDF)
