# Supplementary figures and images for: Notch activation is required for downregulation of HoxA3-dependent endothelial cell phenotype during blood formation
Source: PLoS One. 2017 Oct 26;12(10):e0186818. doi: 10.1371/journal.pone.0186818 (PMC5658089; doi:10.1371/journal.pone.0186818)

Supplementary Figure 1

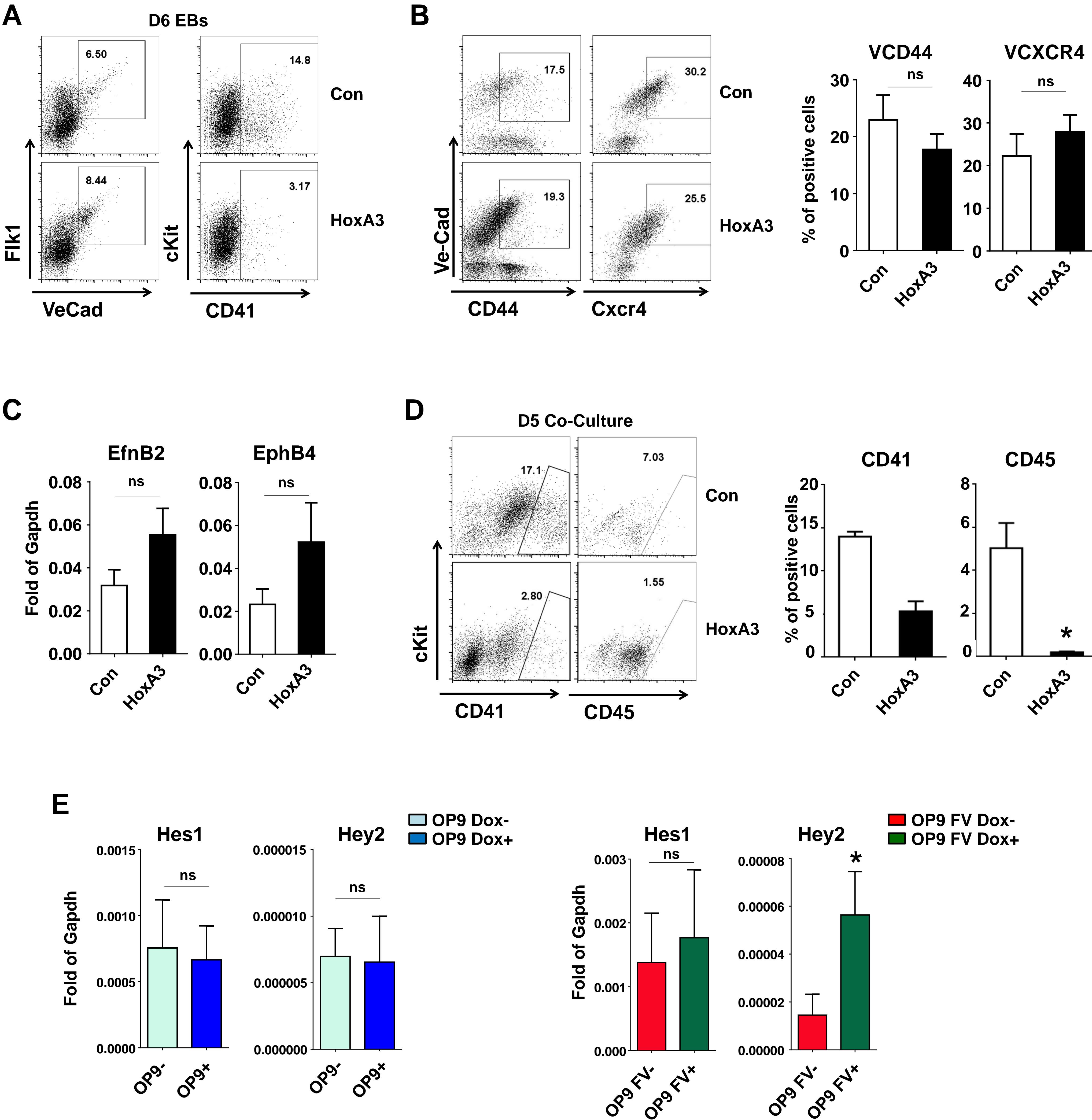

Supplement: S1 Fig — A) Representative flow cytometric profile of endothelial surface markers Flk-1/Ve-Cadherin and hematopoietic surface markers c-Kit/CD41, of 10000 cells obtained from dissociated day 6 EBs treated with or without Dox at day 4 of differentiation. B) Representative flow cytometric profile and frequency quantification of arterial surface markers Ve-Cadherin/CD44 and Ve-Cadherin/CXCR4 on 200,000 EB-derived Flk1+/VE-cadherin+ cells without or with HoxA3 overexpression and co-cultured on OP9 for 5 days. C) Gene expression levels in purified endothelial cells co-cultured with OP9 for 5 day without or with doxycycline-induced HoxA3 overexpression. Arterial, EfnB2 and vein markers EphB4 are plotted. D) Representative flow cytometric profile and frequency quantification of hematopoietic surface markers c-Kit/CD41, and c-Kit/CD45 on 200,000 EB-derived Flk1+/VE-cadherin+ cells without or with HoxA3 overexpression and co-cultured on OP9 for 5 days E) Assessment of Notch pathway activation on OP9 cells alone (left) or purified OP9 cells after co-culture with Flk1+/VE-cadherin+ without or with HoxA3 overexpression (right). Notch target genes Hes1 and Hey2 are plotted. Where present asterisks (*) identify significant paired two-tailed T test (* p<0.05). Statistical analysis is reported on S2 Table. (PDF) [file pone.0186818.s001.pdf]

**Supplementary Figure 2**

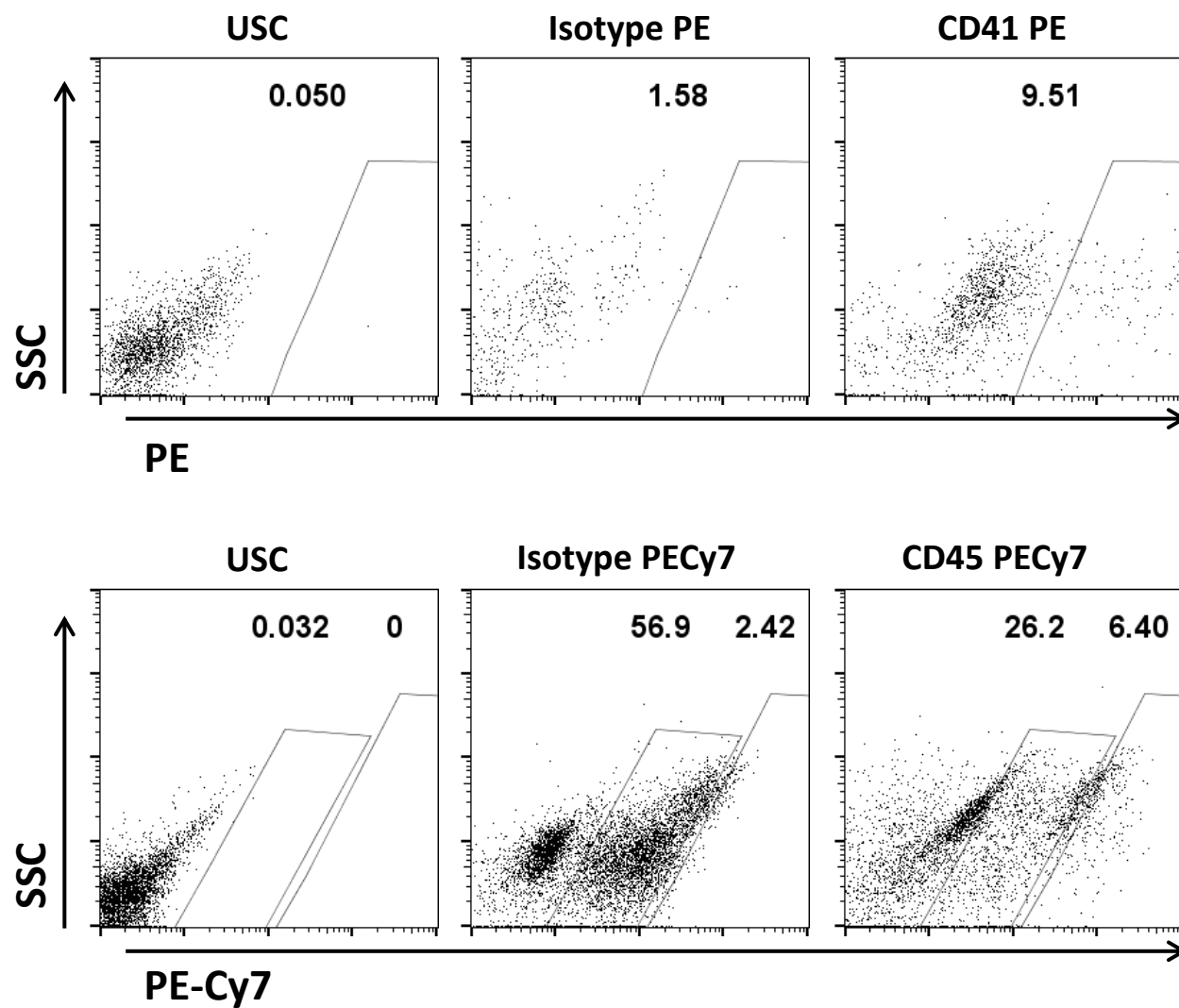

Supplement: S2 Fig — (PDF) [file pone.0186818.s002.pdf]

# Supplementary Figure 3

A

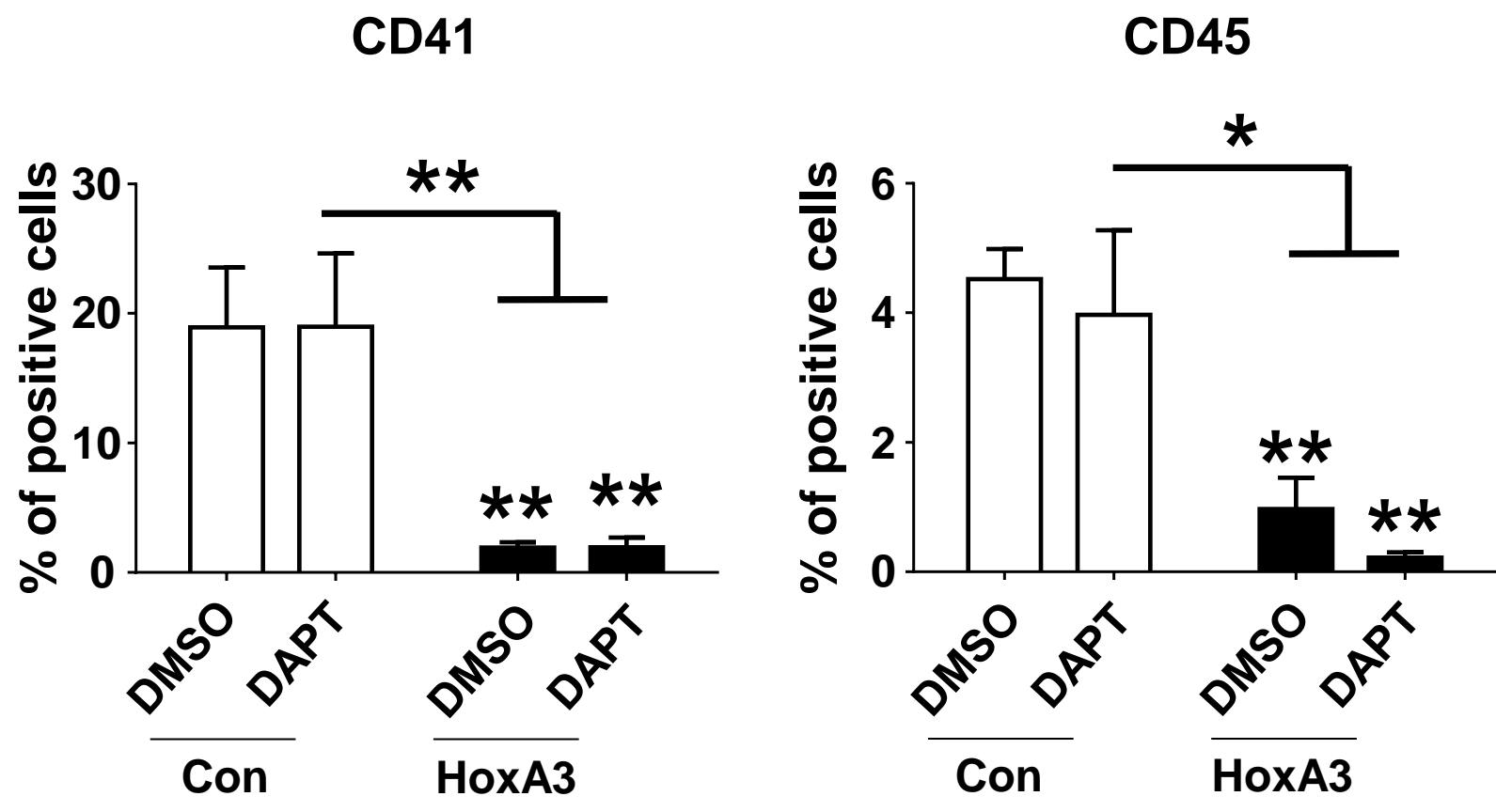

B

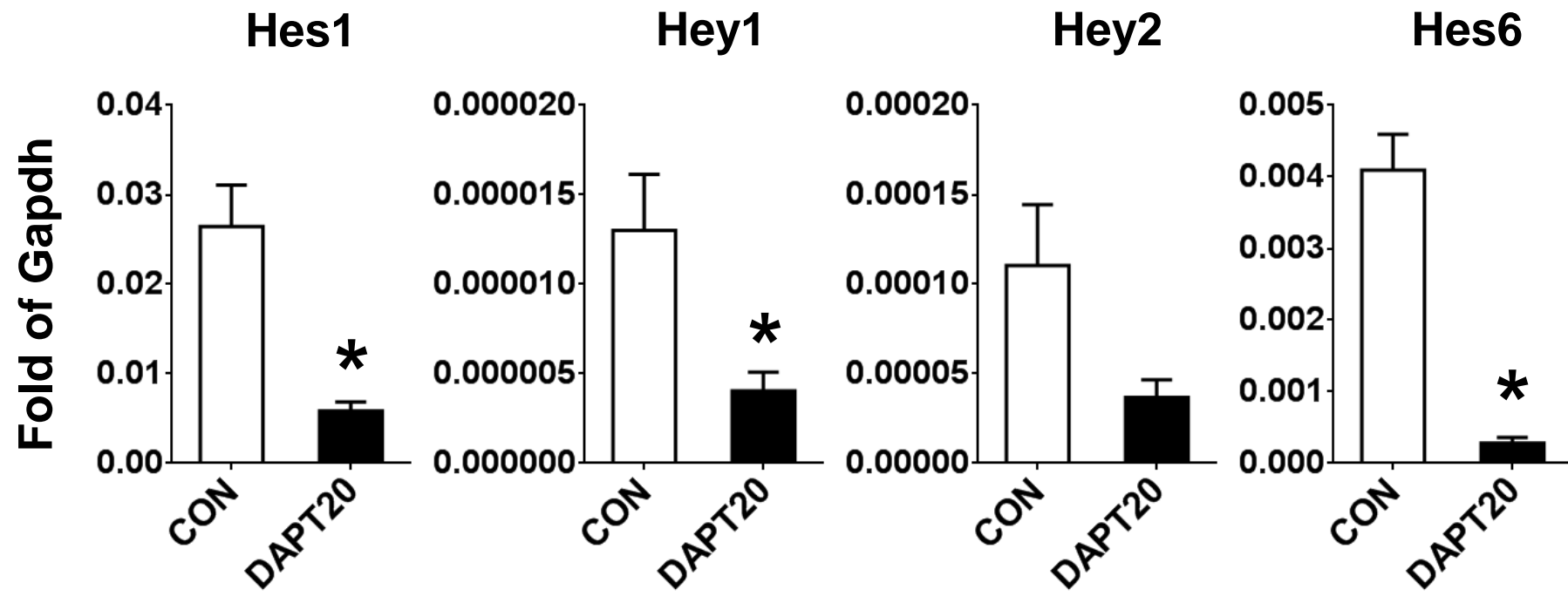

C

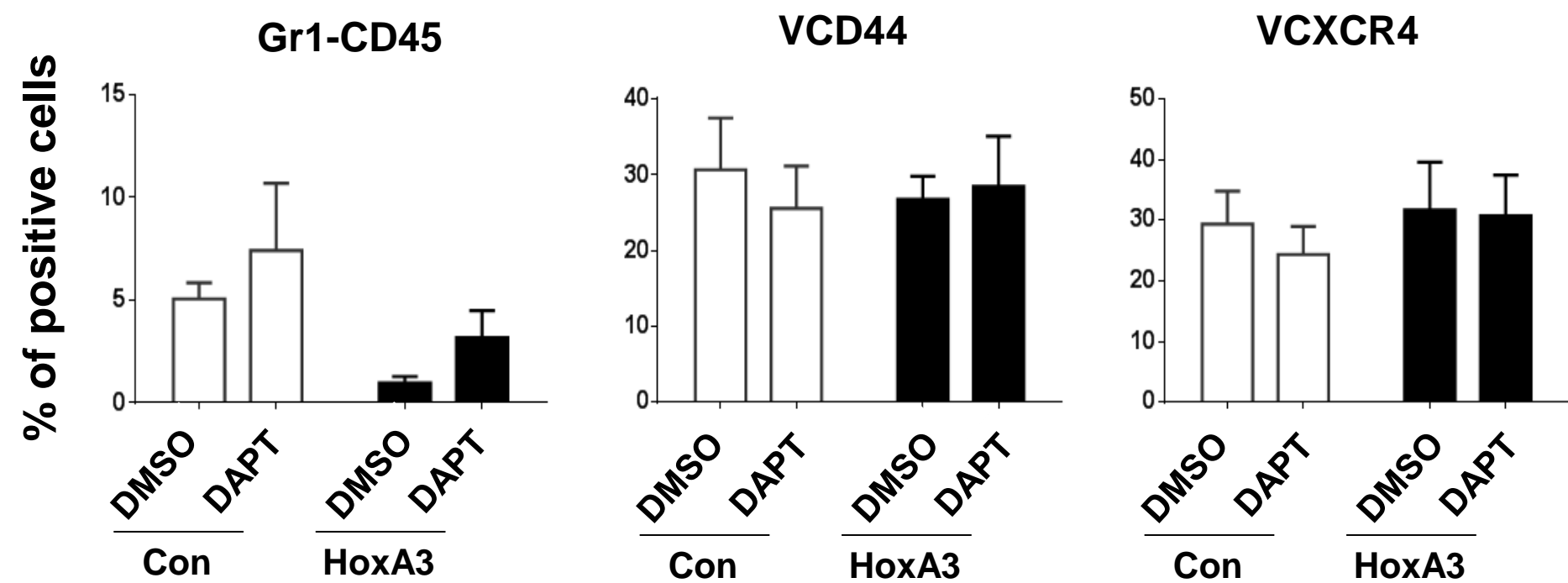

Supplement: S3 Fig — A) Quantification of frequencies of hematopoietic surface markers (ckit-CD41, ckit-CD45) on 200,000 EB-derived Flk1+/VE-cadherin+ cells without or with HoxA3 overexpression and co-cultured on OP9 for 5 days in the presence or absence of the Notch inhibitor DAPT (20μM) B) Evaluation of Notch pathway inhibition (calculated as inhibition of Notch target genes Hes1, Hey1, Hey2, Hes6) on endothelial cells (BEND3) treated with 20μM of DAPT or DMSO (CON). C) Frequency quantification of 200,000 cells Flk1+/VE-cadherin+ obtained from day 6 EBs and co-cultured on OP9 for 5 days with or without HoxA3 overexpression and treated without (DMSO/CON) or with 20μM of DAPT. Hematopoietic surface markers Gr1-CD45 and arterial/vein Ve-Cadherin, CXCR4 and CD44 and are plotted. Statistical analysis is reported on S3 Table. (PDF) [file pone.0186818.s003.pdf]

# Supplementary Figure 4

**A**

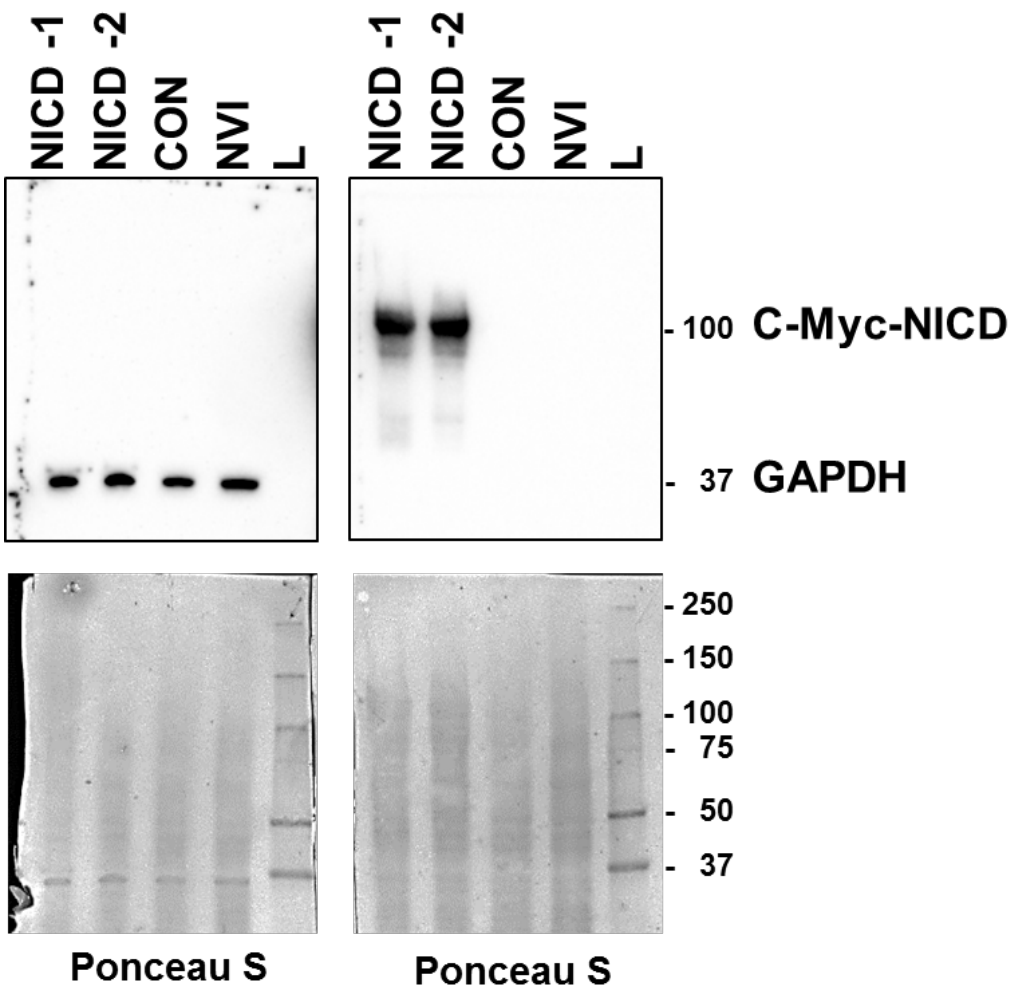

**B**

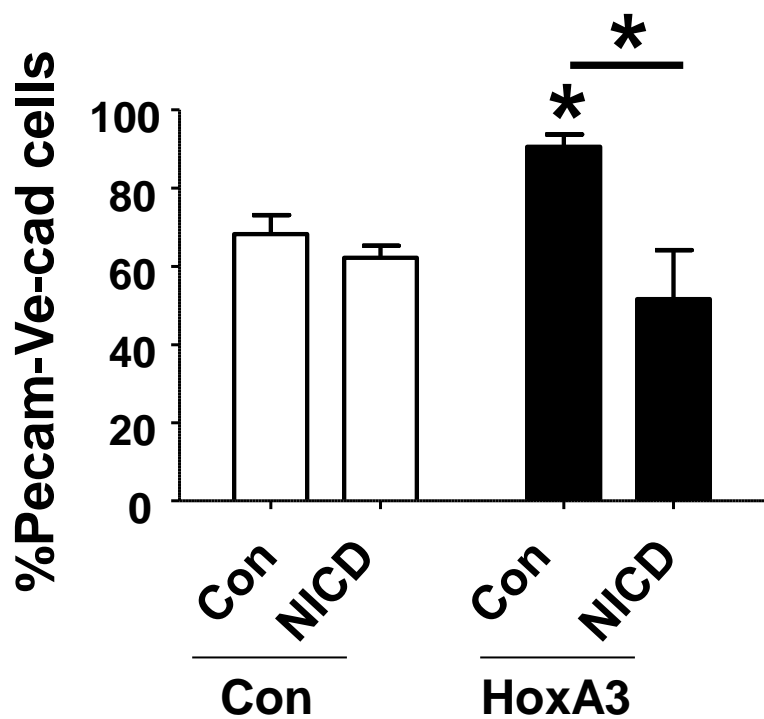

**C**

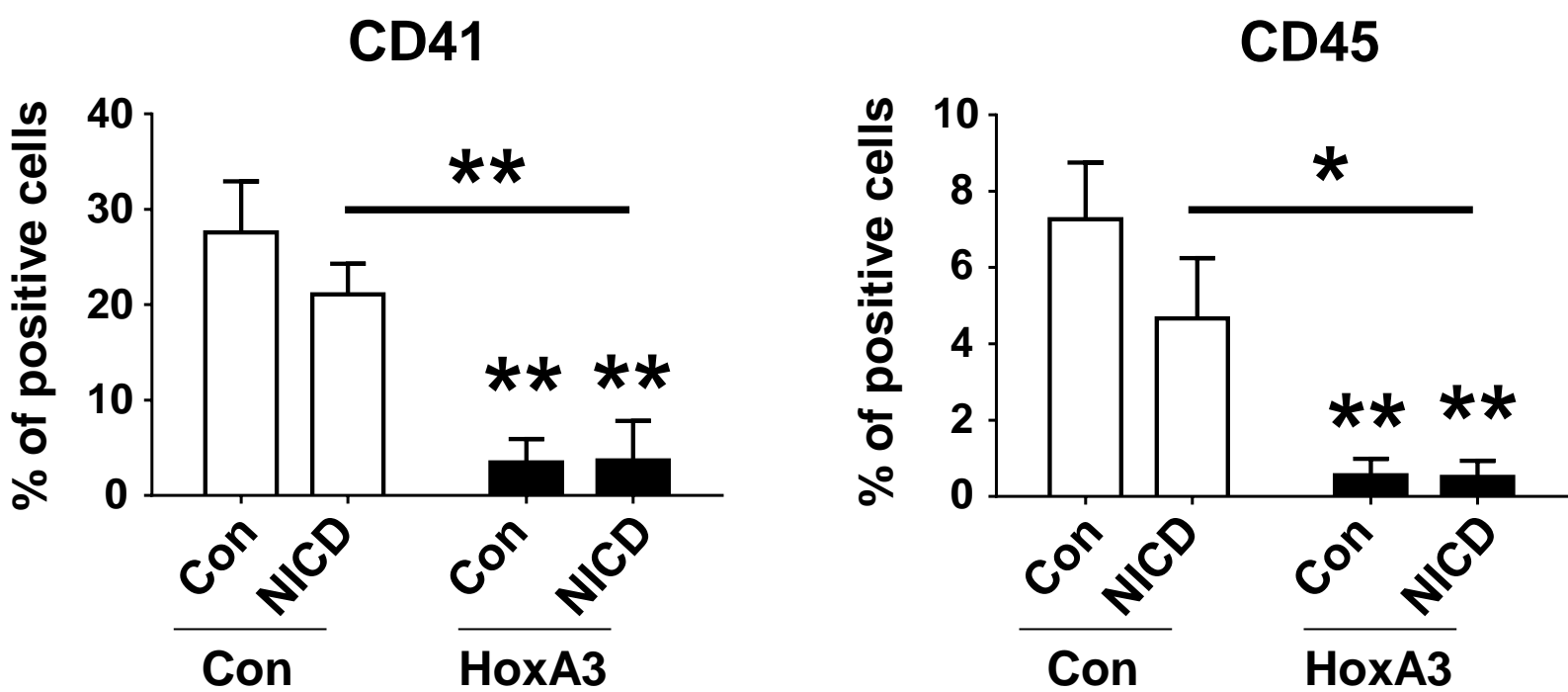

**D**

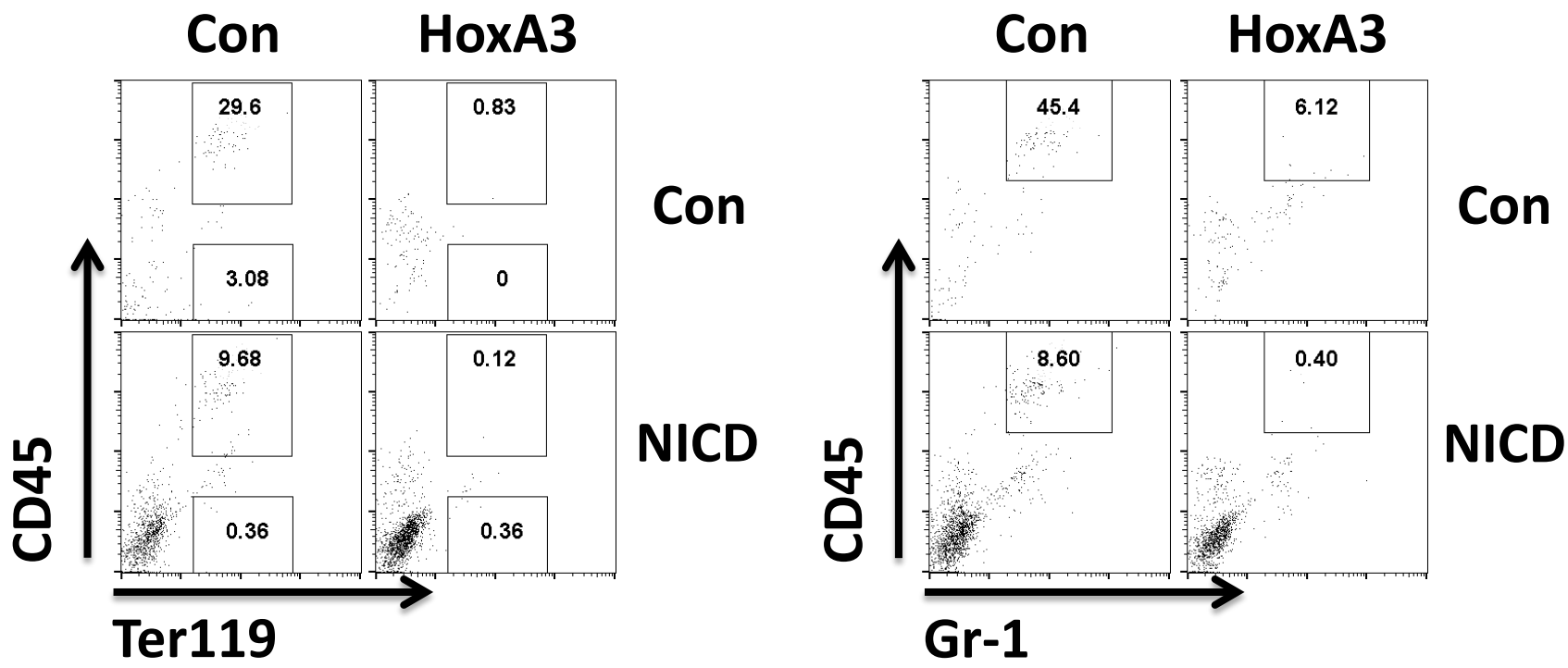

**E**

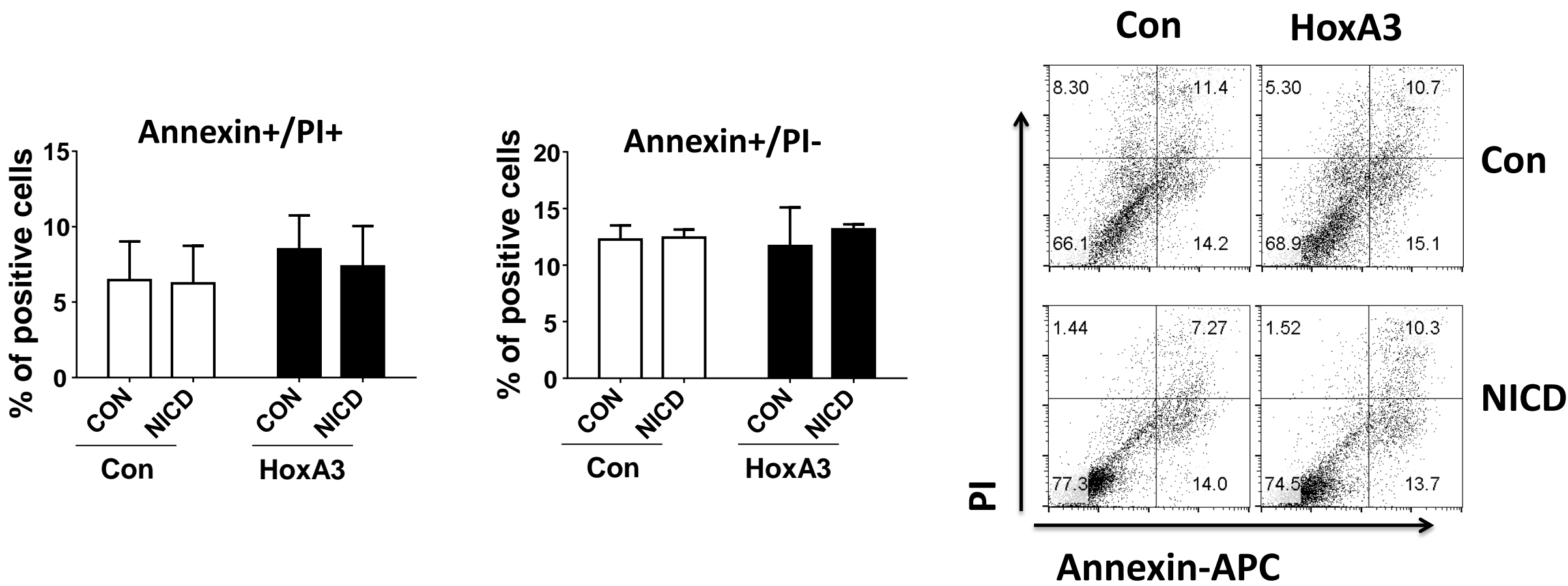

Supplement: S4 Fig — A) Western blot analysis and Ponceau S staining of the indicated proteins (cMyc-NICD and GAPDH) and total loading protein, respectively, in 293T cells transfected with pMSCV-hNICD-ires GFP plasmid (NICD-1/NICD-2), backbone vector pMSCV-ires GFP (CON) and non-viral infection (NVI). B) Frequency quantification of endothelial markers VeCadherin and Pecam (CD31), from gated GFP positive cells transduced with pMSCV-iresGFP (CON) or with pMSCV-hNICD1-IresGFP (NICD) and co-cultured on OP9 for 5 days in absence (CON) or presence (HoxA3) of HoxA3 overexpression. C) Quantification of frequencies of hematopoietic surface markers ckit, CD41, CD45, and D) representative flow cytometric profile of myeloid markers CD45, Gr1 and Ter119 on 200,000 cells Flk1+/VE-cadherin+ obtained from day 6 EBs, transduced with pMSCV-iresGFP (CON) or with pMSCV-hNICD1-IresGFP (NICD) and co-cultured on OP9 for 5 days in absence (CON) or presence (HoxA3) of HoxA3 overexpression. E) Frequency quantification and representative flow cytometric profile, of 200,000 cells Flk1+/VE-cadherin+ obtained from day 6 EBs, transduced with pMSCV-iresGFP (CON) or with pMSCV-hNICD1-IresGFP (NICD) and co-cultured on OP9 for 5 days in absence (CON) or presence (HoxA3) of HoxA3 overexpression. Viability markers PI and Annexin V are plotted. Post-hoc analysis are reported as asterisks (*) alone represents significant differences compared to CON/Dox-, * p<0.05, and bars represents significant differences (*) between indicated groups, p<0.05. Statistical analysis is reported on S4 Table. (PDF) [file pone.0186818.s004.pdf]

# Supplementary Figure 5

Flk1/Ve-Cad

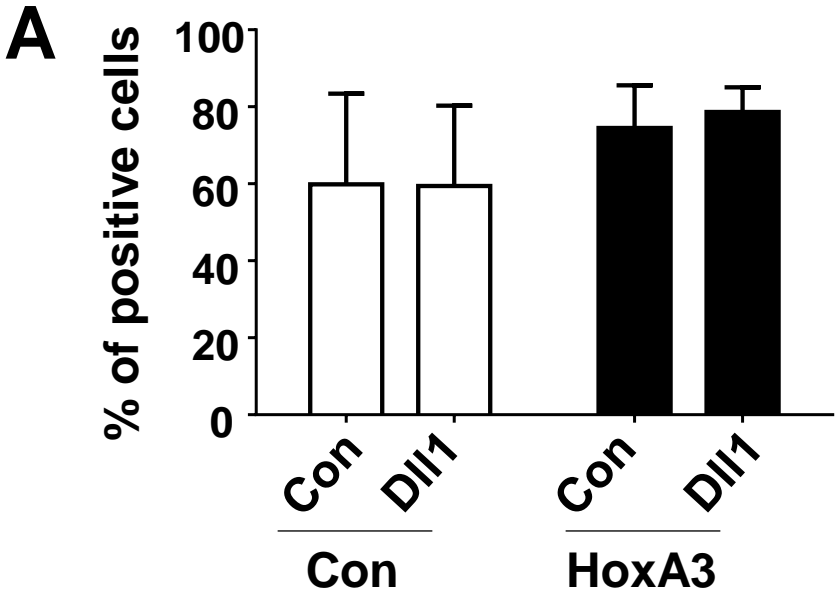

**B**

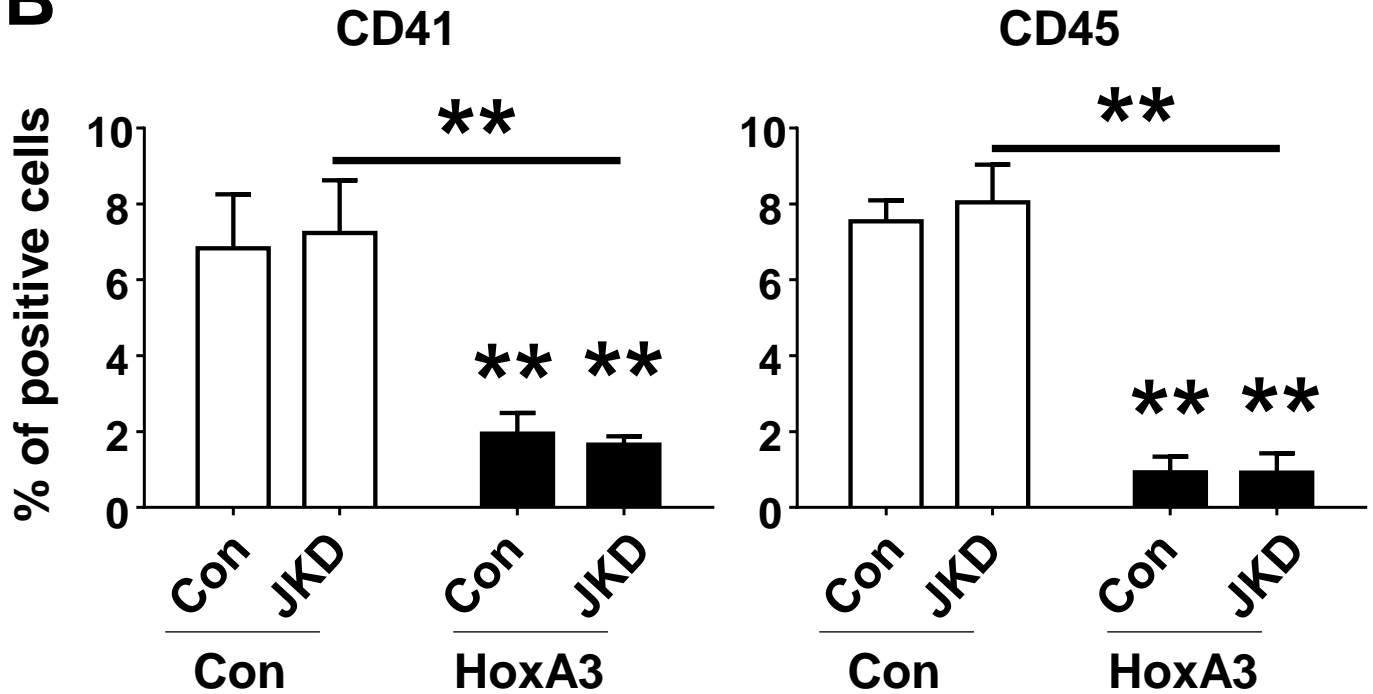

Supplement: S5 Fig — A) Quantification of frequencies of endothelial surface markers Flk-1+/Ve-Cadherin+ obtained from 200,000 EB-derived Flk1+/VE-cadherin+ cells and co-cultured on OP9 control (CON) or OP9 overexpressing Dll1 (OP9-Dll1) for 5 days in Control or HoxA3-overexpressing HE cells. B) Quantification of frequencies of hematopoietic surface markers (cKit-CD41, cKit-CD45) on cells obtained from day 6 EBs, transduced with empty vector (CON) or with shRNA-Jag1-GFP (JKD) and co-cultured on OP9 for 5 days in Control (Con) or HoxA3 overexpression. (PDF) [file pone.0186818.s005.pdf]
