# Supplementary material for: Notch activation is required for downregulation of HoxA3-dependent endothelial cell phenotype during blood formation
Source: PLoS One. 2017 Oct 26;12(10):e0186818. doi: 10.1371/journal.pone.0186818 (PMC5658089; doi:10.1371/journal.pone.0186818)
Supplement: S1 Table — (PDF) [file pone.0186818.s006.pdf]

Table S1

Probes	qPCR	Probes	qPCR
EfnB2	Mm00438670_m1	Jag1	Mm00496902_m1
Dll1	Mm01279269_m1	Jag2	Mm01325629_m1
Dll3	Mm00432854_m1	Lfng	Mm00456128_m1
Dll4	Mm0044619_m1	Mfng	Mm00434941_m1
EphB4	Mm01201157_m1	Notch1	Mm00435249_m1
Gapdh	Mm99999915_g1	Notch2	Mm00803077_m1
Gata1	Mm01352636_m1	Notch3	Mm01345646_m1
Hes1	Mm01342805_m1	Notch4	Mm00440525_m1
Hey1	Mm00468865_m1	Pu.1	Mm00488142_m1
Hey2	Mm00469280_m1	Runx1	Mm01213404_m1
hNotch1	Hs01062014_m1	α-SMA (ACTA2)	Mm00725412_s1
HoxA3	Mm01326402_m1		

Abs	FACs	Abs	FACs
Flk1-PE	555308	Pecam-PE	12-0311-82
CD45-PECy7	25-0451-82	TER119-PE	12-5921-82
c-Kit-APC	17-1171-82	Ve-cadherin-APC	17-1441-8
Gr1-FITC	11-5931-82		
		Isotype-PE	553925
		Isotype-PECy7	25-4031-81

Abs	WB and IF	Abs	WB and IF
Myc-tag	MA1-21316	Goat anti-rabbit 555	A21428
Jag1 H-114	sc8303	Goat anti-rat 488	A11006
Ve-Cad eBioBV13	14-1441-8303		
Activated Notch1	ab8925		
