# Supplementary material for: Notch activation is required for downregulation of HoxA3-dependent endothelial cell phenotype during blood formation
Source: PLoS One. 2017 Oct 26;12(10):e0186818. doi: 10.1371/journal.pone.0186818 (PMC5658089; doi:10.1371/journal.pone.0186818)
Supplement: S2 Table — A) Two tails T-test analysis of Notch components on control endothelial cells (CON) compare to endothelial cells derived from 6 hours upregulation of HoxA3 in D6 total EBs (HoxA3) B) Two tails T-test analysis of Notch components endothelial derived cells (EDC) co-cultured with OP9 for 5 days without (CON) or with HoxA3 overexpression. (PDF) [file pone.0186818.s007.pdf]

Table S2

6h induction	CON				HoxA3				Significance	
	N	Average	±	SE	N	Average	±	SE		
**Jag1**	3	0.023	±	0.007	3	0.085	±	0.013	p= 0.0156	*
**Dll1**	3	0.040	±	0.012	3	0.049	±	0.016		
**Dll3**	3	0.004	±	0.001	3	0.007	±	0.003		
**Hes1**	3	0.003	±	0.000	3	0.002	±	0.000		
**Notch1**	3	0.180	±	0.094	3	0.107	±	0.023		

5 days induction	CON				HoxA3				Significance	
	N	Average	±	SE	N	Average	±	SE		
**Jag1**	7	0.015	±	0.003	7	0.039	±	0.007	p= 0.0156	*
**Jag2**	6	0.002	±	0.001	6	0.000	±	0.000	p= 0.0091	**
**Dll1**	7	0.006	±	0.002	7	0.014	±	0.004	p= 0.0336	*
**Dll3**	4	0.000	±	0.000	4	0.000	±	0.000		
**Dll4**	6	0.002	±	0.001	6	0.000	±	0.000	p= 0.0291	*
**Hes1**	7	0.006	±	0.003	7	0.002	±	0.001		
**Hey2**	7	0.000	±	0.000	7	0.000	±	0.000		
**Hey1**	4	0.002	±	0.002	3	0.002	±	0.000		
**HoxA3**	7	0.001	±	0.001	7	0.081	±	0.029	p= 0.016	*
**Notch1**	6	0.028	±	0.012	6	0.016	±	0.004		
**Notch2**	6	0.000	±	0.000	6	0.000	±	0.000		
**Notch3**	6	0.002	±	0.001	6	0.007	±	0.006		
**Notch4**	6	0.006	±	0.003	6	0.004	±	0.001		
**Lfng**	4	0.006	±	0.002	4	0.001	±	0.000	p= 0.056	#
**Mfng**	4	0.006	±	0.001	4	0.003	±	0.001	p= 0.0393	*
**EfnB2**	4	0.031	±	0.007	4	0.055	±	0.012		
**EphB4**	4	0.02	±	0.01	4	0.05	±	0.02		
**Cd41**	4	14.000	±	0.574	4	5.325	±	1.167	p= 0.0006	***
**Cd45**	4	5.030	±	1.171	4	0.193	±	0.045	p= 0.0062	**
**V-Cd44**	4	0.797	±	6.359	4	22.850	±	3.839		
**V-CXCR4**	4	14.670	±	6.620	4	26.250	±	5.117		
