# Supplementary material for: Notch activation is required for downregulation of HoxA3-dependent endothelial cell phenotype during blood formation
Source: PLoS One. 2017 Oct 26;12(10):e0186818. doi: 10.1371/journal.pone.0186818 (PMC5658089; doi:10.1371/journal.pone.0186818)
Supplement: S3 Table — 2-way ANOVA analysis of endothelial derived cells co-cultured with OP9 for 5 days without (CON) or with HoxA3 overexpression and treated without (DMSO) or with (DAPT) Notch inhibitor. (PDF) [file pone.0186818.s008.pdf]

Table S3

DAPT treatment	CON/DMSO				CON/DAPT				HoxA3/DMSO				HoxA3/DAPT				Anova		
	N	Avg	±	SE	N	Avg	±	SE	N	Avg	±	SE	N	Avg	±	SE	Dox treatment	DAPT treatment	Dox/DAPT interaction
**Ve-Cad**	3	57.63	±	5.12	3	65.43	±	5.72	3	80.37	±	4.34	3	87.67	±	1.60	F(1,8)=69.989 p=0.001		
**Cd41**	3	18.93	±	2.66	3	19.00	±	3.26	3	1.93	±	0.25	3	1.96	±	0.42	F(1,8)=64.55 p<0.0001		
**Cd45**	3	4.52	±	0.46	3	3.97	±	1.31	3	0.97	±	0.49	3	0.22	±	0.08	F(1,8)=24.61 p=0.0011		
