# Supplementary material for: Notch activation is required for downregulation of HoxA3-dependent endothelial cell phenotype during blood formation
Source: PLoS One. 2017 Oct 26;12(10):e0186818. doi: 10.1371/journal.pone.0186818 (PMC5658089; doi:10.1371/journal.pone.0186818)
Supplement: S4 Table — 2-way ANOVA analysis of endothelial derived cells transduced with pMSCV-NICD-ires GFP (NICD) or pMSCV-iresGFP (CON) and co-cultured with OP9 for 5 days without (CON) or with HoxA3 overexpression. (PDF) [file pone.0186818.s009.pdf]

Table S4

	CON				NICD				HoxA3				NICD/hoxA3				Anova		
**NICD**	**N**	**Avg**	**±**	**SE**	**N**	**Avg**	**±**	**SE**	**N**	**Avg**	**±**	**SE**	**N**	**Avg**	**±**	**SE**	**Dox treatment**	**NICD infection**	**Dox/NICD interaction**
**VE-cad**	8	69.86	±	5.25	8	64.91	±	3.28	8	86.90	±	1.88	8	33.15	±	1.99	F(1,28)=4.73 p=0.038	F(1,28)=75.18 p<0.0001	F(1,28)=51.96 p<0.0001
**Cd41**	7	27.58	±	5.37	7	21.09	±	3.20	7	3.46	±	0.94	7	3.69	±	1.56	F(1,28)=40.69 p<0.0001		
**Cd45**	4	7.26	±	1.49	4	4.67	±	1.58	4	0.56	±	0.21	4	0.52	±	0.21	F(1,12)=24.51 p=0.0003		
**HoxA3**	4	0.001	±	0.001	4	0.003	±	0.001	4	0.069	±	0.036	4	0.040	±	0.022	F (1, 12) = 6.204 p=0.0284		
**hNotch1**	4	0.100	±	0.052	4	1.702	±	0.978	4	0.285	±	0.231	4	1.287	±	0.645		F (1, 12) = 4.749 p=0.05	
**Hes1**	4	0.013	±	0.003	4	0.017	±	0.008	4	0.005	±	0.001	4	0.018	±	0.007			
**Hey2**	4	0.000	±	0.000	4	0.038	±	0.013	4	0.001	±	0.000	4	0.044	±	0.017		F (1, 12) = 13.69 p=0.003	
**Hey1**	4	0.003	±	0.002	4	0.033	±	0.017	4	0.004	±	0.002	4	0.029	±	0.010		F (1, 12) = 8.098 p=0.0147	
**PU.1**	4	0.009	±	0.008	4	0.002	±	0.001	4	0.000	±	0.000	4	0.000	±	0.000			
**Gata1**	4	0.002	±	0.001	4	0.004	±	0.002	4	0.000	±	0.000	4	0.000	±	0.000	F (1, 12) = 5.913 p=0.0316		
**Runx1**	3	0.029	±	0.009	3	0.028	±	0.004	3	0.008	±	0.001	3	0.014	±	0.003	F (1, 8) = 10.52 p=0.0118		
**αSMA**	3	0.052	±	0.025	3	0.158	±	0.039	3	0.028	±	0.006	3	0.067	±	0.017	F (1, 8) = 5,369 p=0.0491	F (1, 8) = p=0.0194	
