# Supplementary material for: Notch activation is required for downregulation of HoxA3-dependent endothelial cell phenotype during blood formation
Source: PLoS One. 2017 Oct 26;12(10):e0186818. doi: 10.1371/journal.pone.0186818 (PMC5658089; doi:10.1371/journal.pone.0186818)
Supplement: S5 Table — 2-way ANOVA analysis on endothelial derived cells co-culture with OP9-CON vs OP9-Dll1 for 5 days without (CON) or with HoxA3 overexpression. (PDF) [file pone.0186818.s010.pdf]

Table S5

	OP9/CON				Dll1-Op9/CON				OP9/HoxA3				Dll1-OP9/HoxA3				Anova		
**OP9-DLL1**	**N**	**Avg**	**±**	**SE**	**N**	**Avg**	**±**	**SE**	**N**	**Avg**	**±**	**SE**	**N**	**Avg**	**±**	**SE**	**Dox treatment**	**OP9 coculture effect**	**Dox/OP9 interaction**
**VE-cad**	4	59.82	±	11.75	4	59.42	±	10.43	4	74.47	±	5.53	4	78.60	±	3.19			
**Cd41**	4	13.01	±	2.31	4	23.52	±	3.38	4	1.82	±	0.43	4	1.56	±	0.39	F(1,12)=64.22 p<0.0001	F(1,12)=6.09 p=0.030	F(1,12)=6.73 p=0.023
**Cd45**	4	15.42	±	4.79	4	28.12	±	3.52	4	1.65	±	0.62	4	1.90	±	0.57	F(1,12)=44.37 p<0.0001	F(1,12)=4.65 p=0.052	
**HoxA3**	7	0.001	±	0.000	7	0.001	±	0.001	7	0.034	±	0.011	7	0.053	±	0.013	F (1, 24) = 24.87 p<0.0001		
**Hes1**	7	0.007	±	0.002	7	0.013	±	0.003	7	0.004	±	0.001	7	0.007	±	0.001	F(1, 24)=5.768 p=0.0244	F (1, 24) = 5.66 p=0.0257	
**Hey1**	6	0.002	±	0.001	6	0.006	±	0.002	7	0.002	±	0.001	7	0.005	±	0.001		F (1, 22) = 6.584 p=0.0176	
**Hey2**	5	0.001	±	0.000	5	0.004	±	0.015	5	0.000	±	0.000	5	0.001	±	0.000	F(1, 16)=4.153 p=0.0585	F(1, 16)=4.277 p=0.0552	
