# Supplementary material for: Notch activation is required for downregulation of HoxA3-dependent endothelial cell phenotype during blood formation
Source: PLoS One. 2017 Oct 26;12(10):e0186818. doi: 10.1371/journal.pone.0186818 (PMC5658089; doi:10.1371/journal.pone.0186818)
Supplement: S6 Table — 2-way ANOVA analysis of endothelial derived cells transduced with pGIPZ (CON) or plKO.1-Jag1 KD-ires GFP (JAG KD) and co-cultured with OP9 for 5 days without (CON) or with HoxA3 overexpression. (PDF) [file pone.0186818.s011.pdf]

Table S6

A

JAG KD	CON				JAG KD				HoxA3				JAG KD/HoxA3				Anova		
	N	Avg	±	SE	N	Avg	±	SE	N	Avg	±	SE	N	Avg	±	SE	Dox treatment	JAG KD infection	Dox/OP9 interaction
**VE-cad**	7	61.71	±	8.39	7	52.47	±	6.80	7	73.50	±	6.02	7	51.06	±	6.44		F(1,24)=5.169 p=0.0322	
**Cd41**	7	6.83	±	1.43	7	7.23	±	1.39	7	1.94	±	0.55	7	1.65	±	0.22	F(1,24)=25.41 p<0.0001		
**Cd45**	3	7.55	±	0.55	3	8.04	±	0.99	3	0.93	±	0.41	3	0.92	±	0.50	F(1,8)=110.4 p<0.0001		

B

Jag KD	CON/Dox-				Dll1/Dox-				Dll1 JKD/Dox-				CON/Dox+				Dll1/Dox+				Dll1 JKD/Dox+					
OP9 DLL1	N	Avg	±	SE	N	Avg	±	SE	N	Avg	±	SE	N	Avg	±	SE	N	Avg	±	SE	N	Avg	±	SE	Anova one-way Dox-	Anova one-way Dox+
**Hes1**	8	0.007	±	0.002	8	0.013	±	0.002	3	0.018	±	0.005	8	0.004	±	0.001	8	0.007	±	0.001	3	0.009	±	0.001	F(2, 16)=3.987 p=0.0394	
**Hey1**	7	0.002	±	0.001	8	0.008	±	0.002	3	0.019	±	0.008	8	0.002	±	0.000	8	0.005	±	0.000	3	0.009	±	0.002	F(2, 15)=6.536 p=0.0091	F(2, 16)=11.49 p=0.0008
**Hey2**	6	0.001	±	0.000	6	0.004	±	0.001	3	0.007	±	0.003	6	0.000	±	0.000	6	0.001	±	0.000	3	0.002	±	0.000	F(2, 12)=4.594 p=0.0330	F(2, 12)=20.29 p=0.0001
